# The Effects of Repetitive Use and Pathological Remodeling on Channelrhodopsin Function in Cardiomyocytes

**DOI:** 10.3389/fphys.2021.710020

**Published:** 2021-08-23

**Authors:** Balázs Ördög, Alexander Teplenin, Tim De Coster, Cindy I. Bart, Sven O. Dekker, Juan Zhang, Dirk L. Ypey, Antoine A. F. de Vries, Daniël A. Pijnappels

**Affiliations:** Laboratory of Experimental Cardiology, Department of Cardiology, Leiden University Medical Center, Leiden, Netherlands

**Keywords:** optogenetics, *in vitro*, cellular electrophysiology, action potential, ionic currents

## Abstract

**Aim:** Channelrhodopsins (ChRs) are a large family of light-gated ion channels with distinct properties, which is of great importance in the selection of a ChR variant for a given application. However, data to guide such selection for cardiac optogenetic applications are lacking. Therefore, we investigated the functioning of different ChR variants in normal and pathological hypertrophic cardiomyocytes subjected to various illumination protocols.

**Methods and Results:** Isolated neonatal rat ventricular cardiomyocytes (NRVMs) were transduced with lentiviral vectors to express one of the following ChR variants: H134R, CatCh, ReaChR, or GtACR1. NRVMs were treated with phenylephrine (PE) to induce pathological hypertrophy (PE group) or left untreated [control (CTL) group]. In these groups, ChR currents displayed unique and significantly different properties for each ChR variant on activation by a single 1-s light pulse (1 mW/mm^2^: 470, 565, or 617 nm). The concomitant membrane potential (*V*_m_) responses also showed a ChR variant-specific profile, with GtACR1 causing a slight increase in average *V*_m_ during illumination (*V*_plateau_: −38 mV) as compared with a *V*_plateau_ > −20 mV for the other ChR variants. On repetitive activation at increasing frequencies (10-ms pulses at 1–10 Hz for 30 s), peak currents, which are important for cardiac pacing, decreased with increasing activation frequencies by 17–78% (*p* < 0.05), while plateau currents, which are critical for arrhythmia termination, decreased by 10–75% (*p* < 0.05), both in a variant-specific manner. In contrast, the corresponding *V*_plateau_ remained largely stable. Importantly, current properties and *V*_m_ responses were not statistically different between the PE and CTL groups, irrespective of the variant used (*p* > 0.05).

**Conclusion:** Our data show that ChR variants function equally well in cell culture models of healthy and pathologically hypertrophic myocardium but show strong, variant-specific use-dependence. This use-dependent nature of ChR function should be taken into account during the design of cardiac optogenetic studies and the interpretation of the experimental findings thereof.

## Introduction

Optogenetics uses the light-sensitive proteins as actuators to take control over cellular function (Fenno et al., 2011; Ferenczi et al., 2019). In cardiology, optogenetics has become an important experimental approach during the past decade in the context of both basic and translational research, mostly owing to its unique capacity to precisely and reversibly modulate the membrane potential of cardiomyocytes by the use of microbial rhodopsins. This method has been successfully applied to achieve a wide range of research goals, including cardiac pacing (Arrenberg et al., 2010; Bruegmann et al., 2010), shaping of action potential (AP) waveforms (Park et al., 2014; Govorunova et al., 2016), sympathetic (Yu et al., 2017) and parasympathetic (Moreno et al., 2019; Machhada et al., 2020) neuromodulations of the heart, termination (Bingen et al., 2014), and spatiotemporal control (Majumder et al., 2018) of spiral waves *in vitro*, as well as the termination of ventricular (Bruegmann et al., 2016; Nyns et al., 2017; Li et al., 2021) and atrial fibrillation (Bruegmann et al., 2018; Nyns et al., 2019) *in vivo*. Such great diversity of possible applications has been fueled by the continuous expansion of the optogenetic toolbox with newly identified native microbial rhodopsins and engineered variants of previously identified microbial rhodopsins. Due to these efforts, a greatly diversified set of microbial rhodopsins is now available with distinctly different biophysical properties, such as ion selectivity, gating kinetics, or light absorption spectrum, allowing selection of the optimal variant for a particular application.

Channelrhodopsins (ChRs) are a group of microbial rhodopsins that function as light-gated ion channels. Channelrhodopsin-2 (ChR2) is a blue light-gated cation channel first described in the green alga *Chlamydomonas reinhardtii* (Nagel et al., 2003). ChR2 and its engineered variant carrying the gain-of-function mutation H134R (H134R) (Nagel et al., 2005) are the most frequently used microbial rhodopsin in optogenetic studies (Arrenberg et al., 2010; Bruegmann et al., 2010, 2016, 2018; Park et al., 2014; Moreno et al., 2019). The Ca^2+^-translocating ChR (CatCh) was generated by introducing the L132C mutation into ChR2 (Kleinlogel et al., 2011). Since CatCh possesses increased activation kinetics and light sensitivity compared with ChR2, it has been the microbial rhodopsin of choice in several previous studies in cardiac optogenetics (Bingen et al., 2014; Feola et al., 2017; Watanabe et al., 2017; Majumder et al., 2018). Since the longer wavelengths of visible light penetrate the biological tissues better than the shorter wavelengths, the red-activatable ChR (ReaChR) (Lin et al., 2013), which is excited with orange to red light (590–630 nm), has been the ChR of choice in many *in vivo* studies (Nyns et al., 2017, 2019; Men et al., 2020). In contrast to cation ChRs, anion ChRs, such as the one first isolated in the cryptophyte *Guillardia theta* (GtACR1) (Govorunova et al., 2015), mainly conduct Cl^−^ ions. Given the distinctly different charge carriers, anion ChRs provide an alternative mechanism for controlling the membrane potential (*V*_m_) in cardiomyocytes (Govorunova et al., 2016; Kopton et al., 2018). Considering their prevalence in the literature on cardiac optogenetics and their unique features, these four ChR variants have been included in this study.

To date, microbial rhodopsin variants have been characterized mainly in permanent (non-cardiac) cell lines (e.g., human embryonic kidney 293), *Xenopus laevis* oocytes, or primary neuronal cells to guide the selection of the best-suited variant for a particular application. Comparative data on the performance of different ChRs in cardiomyocytes have not yet been published, leaving the cardiac research community without proper selection guides. Furthermore, in the translational context, one aims to apply optogenetics in the pathological setting, while the ChR of choice should continue to perform well even after extensive and repetitive use. Dedicated and detailed studies into this matter are also lacking. Therefore, in this study, the functional properties of the aforementioned four ChR variants are investigated side-by-side in cardiomyocytes, under normal and pathological conditions while being subjected to illumination protocols of varying duration and intensiveness.

## Materials and Methods

Additional experimental procedures are described in the Supplementary Material.

### Neonatal Rat Ventricular Cardiomyocytes

Neonatal rat ventricular cardiomyocytes (NRVMs) were isolated as described in detail in previous studies (Askar et al., 2013; Bingen et al., 2013). Two-day-old Wistar rats were anesthetized by 5% of isoflurane inhalation, and adequate anesthesia was confirmed by the absence of reflexes. The use of these animals for this particular purpose was approved by the Animal Experiments Committee of the Leiden University Medical Center (AVD116002017818) and conformed to the Guide for the Care and Use of Laboratory Animals as stated by the US National Institutes of Health.

### Molecular Cloning and Lentiviral Vector Production

**Figure 1:**
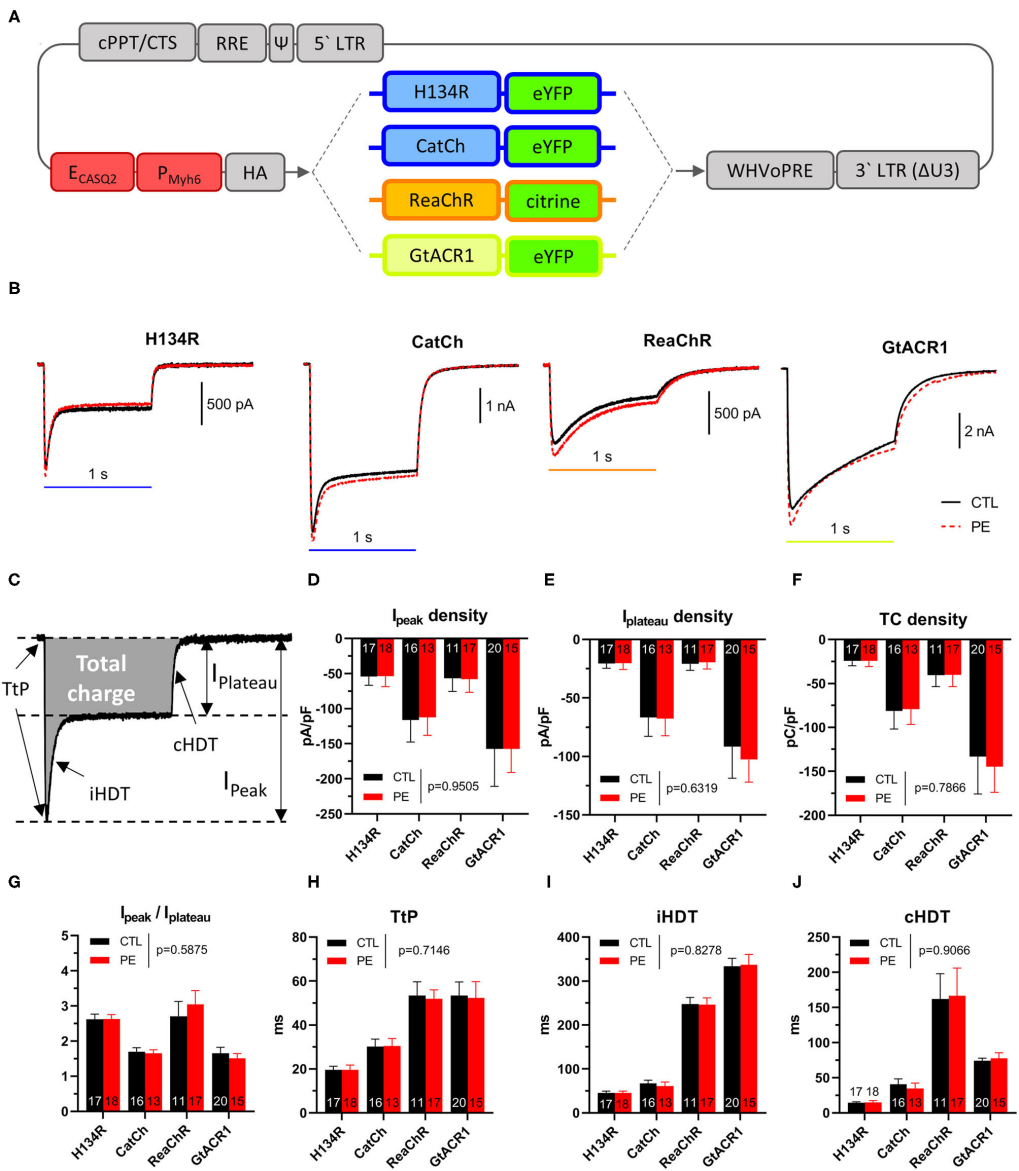
**(A)** Schematic map of lentiviral vector shuttle constructs used in this study. Gray indicates viral vector elements, including the human immunodeficiency virus type 1, 5′ and 3′ long terminal repeats [5′ LTR, 3′ LTR (ΔU3)], packaging signal (Ψ), Rev-response element, central termination site and polypurine tract (cPPT/CTS), the woodchuck hepatitis virus posttranscriptional regulatory element (WHVoPRE), a chimeric cardiomyocyte-specific promoter (dark red) consisting of the enhancer of the human calsequestrin 2 gene (E_CASQ2_) and the promoter of the mouse myosin heavy chain 6 gene (P_Myh6_), the coding sequence of influenza A virus hemagglutinin (HA; N-terminal), and fluorescent protein-tagged (eYFP or citrine, C-terminal) ChR variants (H134R, CatCh, ReaChR, and GtACR1). **(B–J)** Characterization of photocurrents produced by dark-adapted ChR variants after activation by blue (470 nm, for H134R and CatCh), amber (565 nm, for GtACR1) or red (617 nm, for ReaChR) light (1 s, 1 mW/mm^2^) in control (CTL) and in phenylephrine (PE)-treated neonatal rat ventricular cardiomyocytes (NRVMs). (**B**) Representative photocurrent recordings from CTL (black) and PE-treated (red) cells expressing ChR variants. **(C)** Schematic representation of the quantified parameters. **(D)** Peak current (*I*_peak_) densities. **(E)** Plateau current densities (*I*_plateau_). **(F)** Total charge densities. **(G)** *I*_peak_−*I*_plateau_ ratios. **(H)** Time to peak current (TtP). **(I)** Inactivation half-decay time (iHDT). **(J)** Closing half-decay time (cHDT). Data are presented as mean ± 95% CI for the number of cells indicated in the bar graphs. The *p*-values corresponding to the effects of PE treatment vs. CTL conditions were calculated by using two-way ANOVA.

The lentiviral vector (LV) shuttle constructs encoding influenza A virus hemagglutinin (HA) epitope- and fluorescent protein-tagged ChR variants were constructed by standard cloning procedures using plasmids pAAV-hSyn-hChR2(H134R)-EYFP (RRID:Addgene_26973), pLenti-CaMKIIa-hChR2(L132C)-EYFP-WPRE (Feola et al., 2017), pLenti-ReaChR-citrine (RRID:Addgene_50956) (Lin et al., 2013), and pFUG-hGtACR1-EYFP (RRID:Addgene_67795) as starting materials (Figure 1A). Self-inactivating LV particles were produced in 293T cells and were purified as previously described (Feola et al., 2016). The functional titer of each LV batch was determined by limiting dilution assays in NRVM monolayers and was taken into consideration during transductions to ensure equal LV dose in each experimental group. A single batch of each LV vector was used for the entire study to eliminate any possible experimental variability arising from the use of vector batches of different titer purity and/or quality.

### Cell Culture and Transduction

Neonatal rat ventricular cardiomyocytes were seeded on bovine fibronectin (Sigma-Aldrich)-coated culture plates or round coverslips and were kept in Advanced Dulbecco's Modified Eagle Medium supplemented with 2% of heat-inactivated horse serum, GlutaMAX Supplement (2 mM), and Penicillin-Streptomycin (50 U and 50 μg/ml, respectively, all from Thermo Fisher Scientific). Fibroblast growth was suppressed by mitomycin-C treatment (Askar et al., 2011) on Day 2. On the same day, NRVMs were transduced with equal doses of each LV at a multiplicity of infection of 1.5, which resulted in ~80% yellowish-green fluorescent cells as assessed by fluorescence microscopy. Hypertrophy was induced by exposing the NRVMs for 24 h to 100 μM of phenylephrine (PE) on Day 3. PE-treated NRVMs showed hallmarks of pathological hypertrophy, including significantly (*p* < 0.05) increased cell surface area, atrial natriuretic peptide level, total protein content, and AP duration (APD) compared with control (CTL) cells as early as on Day 5 (Supplementary Figure 1).

### Patch Clamp and Light Delivery

Patch clamp experiments were carried out at room temperature (23–25°C) on single, well-separated NRVMs cultured on round coverslips in 24-well plates at a density of 10^4^ cells/well. The measurements took place in yellowish-green fluorescent NRVMs on Days 5 and 6, using the perforated and ruptured patch clamp configurations for the recording of *V*_m_ and transmembrane currents, respectively, as described in detail in the Supplementary Material.

To activate ChRs, NRVMs were illuminated by using commercially available 470-, 565- or 617-nm collimated light-emitting diodes (LEDs) (Thorlabs, Newton, NJ, United States) mounted on three independent camera ports of a Zeiss Axiovert 35 inverted microscope *via* a 40× magnification objective lens. LEDs were controlled by using Clampex software (Molecular Devices, San Jose, CA, United States) using independent analog outports of the Digidata 1440 D/A interface (v10, Molecular Devices, San Jose, CA, United States). The same illumination intensity (1 mW/mm^2^) was used in all experiments and for all wavelengths.

### Data Analysis and Statistical Methods

Numeric data were extracted from Axon Binary Format files by using the pyABF module^1^ and were analyzed using custom software written in house dedicated to each experimental type, as described in detail in the Supplementary Material. Statistical analyses were carried out by using GraphPad Prism software (version 8.4.2, Graphpad Software, LLC, San Diego, CA, United States). In most of the analyses, group means were compared by using the two-way ANOVA, considering “treatment” (i.e., PE and CTL) and “ChR variant” (i.e., H134R, CatCh, ReaChR, and GtACR1) or “treatment” and “preconditioning frequency” (1, 2, 3, 4, 5, 7.5, and 10 Hz) as independent discrete variables and using Tukey's *post-hoc* analysis to correct for multiple comparisons. Preconditioned current parameters were normalized to the corresponding dark-adapted values obtained from the same cell, yielding relative preconditioned parameters. The one-sample Student's *t*-test was used to test whether relative 1-Hz preconditioned values differed from the corresponding dark-adapted values represented by the hypothetical value of 1. To assess the “ChR variant” effect on relative 1-Hz preconditioned current parameters, 2–10-Hz preconditioning data were omitted, and the two-way ANOVA was carried out on 1-Hz preconditioned values using “ChR variant” and “treatment” as independent variables. Linear regression was used to detect preconditioning frequency-dependent changes by testing whether non-zero slopes were present. A possible effect of “treatment” or “ChR variant” on slopes was assessed in follow-up tests using two-way ANOVA. A *p* < 0.05 was considered statistically significant. Numerical data are presented as mean and the 95% CI in the form of [lower boundary, upper boundary], and *n* represents the number of experiments from independent cells.

## Results

### Dark-Adapted Photocurrent Properties

Channelrhodopsin currents are known to undergo inactivation (also known as desensitization) due to use-dependent transitions between dark- and light-adapted states and to recover from inactivation on the seconds time scale (Nagel et al., 2003; Schneider et al., 2015). When ChRs are activated repetitively, partial recovery from inactivation results in decreased photocurrent amplitudes. In order to prevent such bias, in the first set of experiments, ChRs were allowed to undergo complete dark adaptation. To this end, NRVMs expressing ChR variants were kept in the dark for 3 min before the application of each light pulse. Cells were then illuminated by a single, 1-s light pulse of 1 mW/mm^2^. H134R and CatCh were activated by blue (470 nm) light, while GtACR1 and ReaChR were opened with amber (565 nm) and red (617 nm) light, respectively.

Whole-cell photocurrents showed distinct characteristics for each of the four ChR variants, with similar dynamics observed in CTL and in PE-treated NRVMs (Figure 1B). For each ChR variant, the maximal current (*I*_peak_) density was reached shortly after light onset with the time to *I*_peak_ (TtP, Figure 1C) being indicative for the opening speed of a particular ChR variant. After reaching *I*_peak_, photocurrent amplitudes decayed to the so-called plateau current (*I*_plateau_), which was defined as the current amplitude at the end of the light pulse. The rate of current decay from *I*_peak_ to *I*_plateau_ was characterized by the inactivation half-decay time (iHDT). Following light offset, currents returned to baseline with kinetics distinctly different for each ChR variant, characterized as closing half-decay time (cHDT).

Quantitative properties of dark-adapted photocurrents are shown in Table 1 and Figures 1D–J. *I*_peak_ did not differ between CTL and PE-treated NRVMs (*p* = 0.9505, Figure 1D). GtACR1 produced the largest *I*_peak_, followed by CatCh (*p* = 0.0298). *I*_peak_ of ReaChR and H134R was similar but smaller than that of CatCh (*p* = 0.034 and *p* = 0.0007 compared with CatCh, respectively). Similar to *I*_peak_, *I*_plateau_ did not significantly differ between PE-treated and CTL cells (*p* = 0.6319, Figure 1E). NRVMs expressing GtACR1 exhibited the largest *I*_plateau_, followed by CatCh (*p* = 0.0007), ReaChR (*p* < 0.0001), and H134R (*p* < 0.0001). ReaChR and H134R *I*_plateau_ densities were not different (*p* > 0.9999). GtACR1 and CatCh conduct the largest and second largest amount of cell size-normalized total charge (TC), respectively (*p* < 0.0001) (Figure 1F). TC densities were not different (*p* = 0.5191) between ReaChR and H134R but much smaller than those of GtACR1 and CatCh (*p* < 0.05, Figure 1F). PE administration did not affect TC densities (*p* = 0.7866, Figure 1F). Interestingly, *I*_peak_−*I*_plateau_ ratios were similar for CatCh and GtACR1 (*p* > 0.05) but were lower compared with those of H134R and ReaChR (*p* < 0.05) (Figure 1G), which can be explained by the relatively low level of CatCh and GtACR1 inactivation (Figure 1B).

**Table 1:** Dark-adapted photocurrent properties in control (CTL) and phenylephrine (PE)-treated NRVMs expressing H134R, CatCh, ReaChR, or GtACR1.

	**CTL**	**PE**
	**I**_peak_ **(pA/pF)**	
H134R	−54.4 [−66.8, −41.9], *n* = 17	−54.2 [−68.8, −39.5], *n =* 18
CatCh	−116.2 [−147.7, −84.7], *n =* 16	−112.5 [−138.2, −86.9], *n =* 13
ReaChR	−57 [−75.5, −38.4], *n =* 11	−58.2 [−76.7, −39.7], *n =* 17
GtACR1	−157.5 [−210.6, −104.3], *n =* 20	−157.4 [−191, −123.9], *n =* 15
	**I**_plateau_ **(pA/pF)**	
H134R	−20.5 [−24.7, −26.3], *n =* 17	−20.4 [−25.7, −15.1], *n =* 18
CatCh	−66.8 [−82.8, −50.7, *n =* 16]	−67.8 [−82.3, −53.2, *n =* 13]
ReaChR	−20.9 [−26.5, −15.2], *n =* 11	−19.5 [−25.3, −13.5], *n =* 17
GtACR1	−91.8 [−118.6, −64.9, *n =* 20]	−102.7 [−122, −83.4], *n =* 15
	**Total charge (pC/pF)**	
H134R	−24.5 [−29.9, −19.1], *n =* 17	−24.4 [−30.8, −18.1], *n =* 18
CatCh	−81.3 [−101.9, −60.7], *n =* 16	−79.4 [−96.6, −62.2], *n =* 13
ReaChR	−40.8 [−53.6, −28], *n =* 11	−40.2 [−53.6, −26.8], *n =* 17
GtACR1	−133.2 [−175.7, −90.8], *n =* 20	−144.7 [−173.8, −115.6], *n =* 15
	**I**_peak_ **/I**_plateau_	
H134R	2.6 [2.5, 2.8], *n =* 17	2.6 [2.5, 2.6], *n =* 18
CatCh	1.7 [1.6, 1.8], *n =* 16	1.6 [1.5, 1.8], *n =* 13
ReaChR	2.7 [2.3, 3.1], *n =* 11	3 [2.6, 3.4], *n =* 17
GtACR1	1.6 [1.5, 1.8], *n =* 20	1.5 [1.4, 1.6], *n =* 15
	**Time to peak (ms)**	
H134R	19.6 [17.9, 21.2], *n =* 17	19.5 [17.3, 21.8], *n =* 18
CatCh	30.1 [26.7, 33.6], *n =* 16	30.5 [27.1, 33.9], *n =* 13
ReaChR	53.4 [47.2, 59.7], *n =* 11	51.9 [47.7, 56], *n =* 17
GtACR1	53.3 [47.1, 59.6], *n =* 20	52.3 [44.9, 59.7], *n =* 15
	**Inactivation half-decay time (ms)**	
H134R	45.7 [41.7, 49.7], *n =* 17	45.7 [41.8, 49.6], *n =* 18
CatCh	67 [59.6, 74.4], *n =* 16	60.8 [51.4, 70.1], *n =* 13
ReaChR	247.6 [232.7, 262.6], *n =* 11	246.4 [231.3, 261.5], *n =* 17
GtACR1	333.7 [315.5, 351.8], *n =* 20	337 [313.4, 360.6], *n =* 15
	**Closing half-decay time (ms)**	
H134R	14.4 [13, 15.9], *n =* 17	14.7 [11.9, 17.5], *n =* 18
CatCh	40.5 [32.7, 48.3], *n =* 16	34.6 [26.7, 42.4], *n =* 13
ReaChR	161.9 [126.1, 197.8], *n =* 11	166.8 [127.7, 205.8], *n =* 17
GtACR1	74.1 [70.3, 77.8], *n =* 20	77.7 [70, 85.3], *n =* 15

*Data are presented as mean and 95% CI, n represents the number of cells*.

Then, the kinetic properties (i.e., TtP, iHDT, and cHDT) of dark-adapted ChR currents were determined for each ChR variant both in CTL and in PE-treated NRVMs (Table 1 and Figures 1H–J). Pathological hypertrophy of NRVMs, as induced by PE, had no significant effect on TtP, iHDT, or cHDT (*p* = 0.7654, *p* = 0.8278, and *p* = 0.9066, compared with CTL, respectively). However, each variant exhibited distinctive kinetic properties. For example, H134R exhibited the smallest average TtP, iHDT, and cHDT and was therefore the fastest ChR variant (*p* < 0.05, Figures 1H–J). CatCh was slower compared with H134R (*p* < 0.05), but faster than ReaChR and GtACR1 (*p* < 0.05) with respect to all three kinetic parameters. ReaChR and GtACR1 showed similar TtP values (*p* = 0.9999) and required a prolonged period to reach *I*_peak_ following the activation by light (Figure 1H). However, ReaChR was inactivating faster compared with GtACR1 (*p* < 0.0001, Figure 1I), whereas GtACR1 was closing much faster than ReaChR (*p* < 0.0001, Figure 1J).

### Membrane Potential (*V*_m_) Response to Dark-Adapted ChR Activation

**Figure 2:**
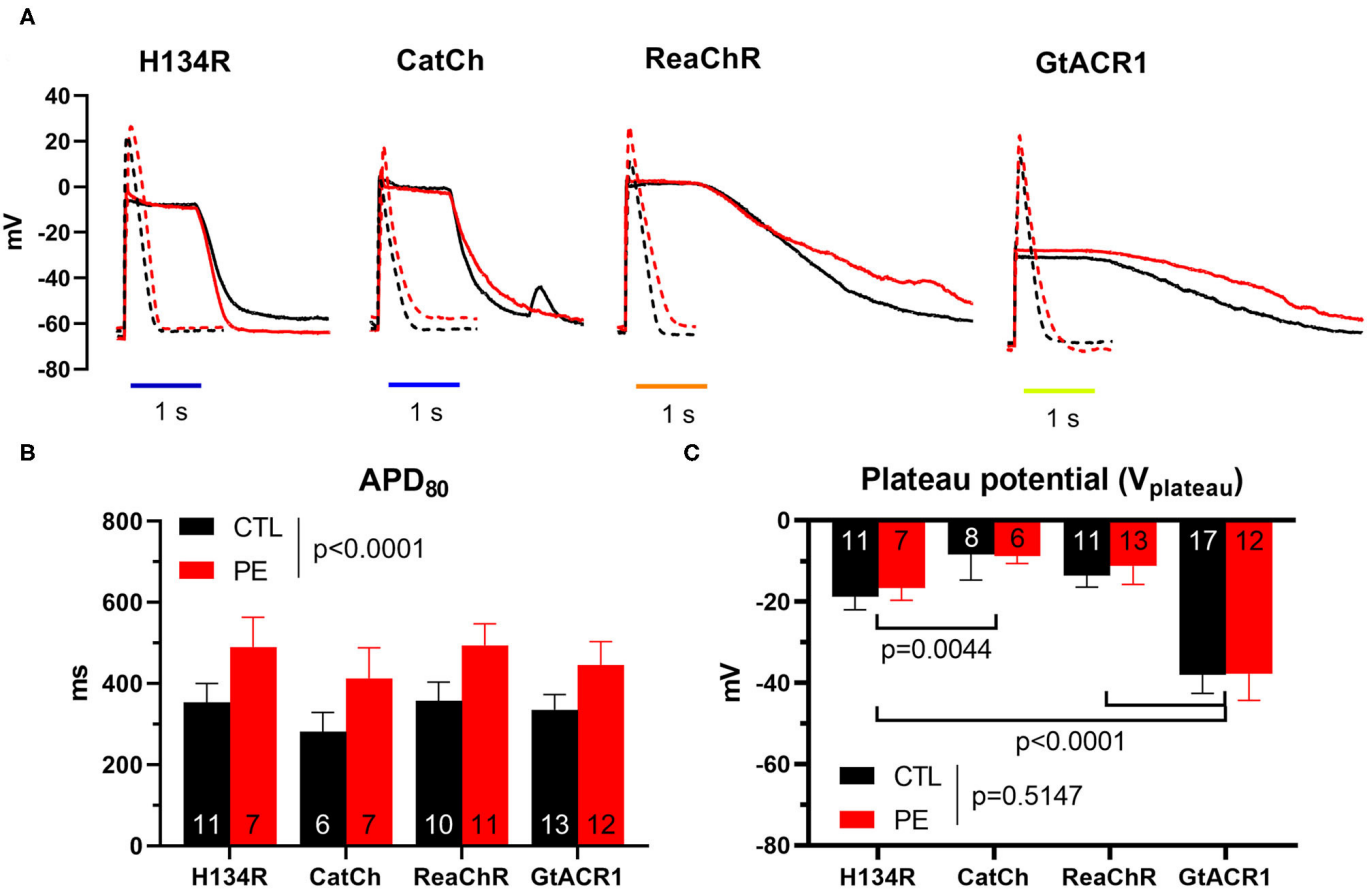
Membrane potential (*V*_m_) responses of CTL and PE-treated NRVMs expressing one of the dark-adapted ChR variants, elicited by electrical pacing at 1 Hz or illumination by blue (470 nm, for H134R and CatCh), amber (565 nm, for GtACR1), or red (617 nm, for ReaChR) light (1 s, 1 mW/mm^2^). **(A)** APs (dashed) and light-induced *V*_m_ response (solid) in CTL (black) and in PE-treated (red) cells. **(B)** Action potential durations (APDs), represented by APD_80_ values. **(C)** Plateau potential (*V*_plateau_) values, defined as *V*_m_ at the end of the illumination. Data are presented as mean ± 95% CI for the number of cells indicated in the bar graphs. The *p*-values corresponding to the effects of PE treatment vs. CTL conditions were calculated by using two-way ANOVA.

The *V*_m_ response to dark-adapted ChR activation was studied by the perforated patch clamp technique under the previously used illumination regime, using a single, 1-s light pulse of 1 mW/mm^2^, following a dark period of 3 min to allow complete ChR recovery from inactivation. For the cation ChRs (i.e., H134R, CatCh, and ReaChR) and the anion ChR (i.e., GtACR1), illumination resulted in pronounced membrane depolarization (Figure 2A). During the dark period, electrically triggered APs were recorded, showing PE-induced prolongation of APD at 80% repolarization (APD_80_) as a sign of electrical remodeling (*p* < 0.0001, Figure 2B). The effect of ChR activation on the membrane potential was characterized by measuring *V*_m_ at the time of light offset, yielding a parameter that is often referred to as the plateau potential (*V*_plateau_). Despite the marked electric remodeling of PE-treated NRVMs (Figure 2B), average *V*_plateau_ values were not statistically different in CTL compared with PE-treated cells (*p* = 0.5147, Figure 2C). GtACR1 activation resulted in the most negative average *V*_plateau_ (−38 mV, 95% CI [−42.6, −33.4] in CTL and −37.8 mV, 95% CI [−44.3, −31.3] in PE-treated cells). Significantly, more positive *V*_plateau_ values were achieved with H134R and ReaChR (*p* < 0.0001), as H134R activity resulted in −18.8 mV (95% CI [−22.0, −15.7]) and −16.7 mV (95% CI [−19.7, −13.6]) *V*_plateau_ values on average, whereas ReaChR activation depolarized the cell membrane to −13.5 mV (95% CI [−16.4, −10.7]) and −11.3 mV (95% CI [−15.8, −6.7]) in CTL vs. PE-treated NRVMs, respectively (Figure 2C). Illumination gave rise to the most robust membrane depolarization in the presence of CatCh (*p* = 0.0044 vs. H134R), resulting in −8.3 mV (95% CI [−14.7, −2.0]) and −8.9 mV (95% CI [−10.6, −6.9]) average *V*_plateau_ values in CTL and PE-treated NRVMs, respectively (Figure 2C). Despite the varying degree of light-induced membrane depolarization, each ChR variant prevented the initiation of APs by electrical stimulation during illumination (Supplementary Figure 2).

### Photocurrent Properties During Preconditioning by Light

**Figure 3:**
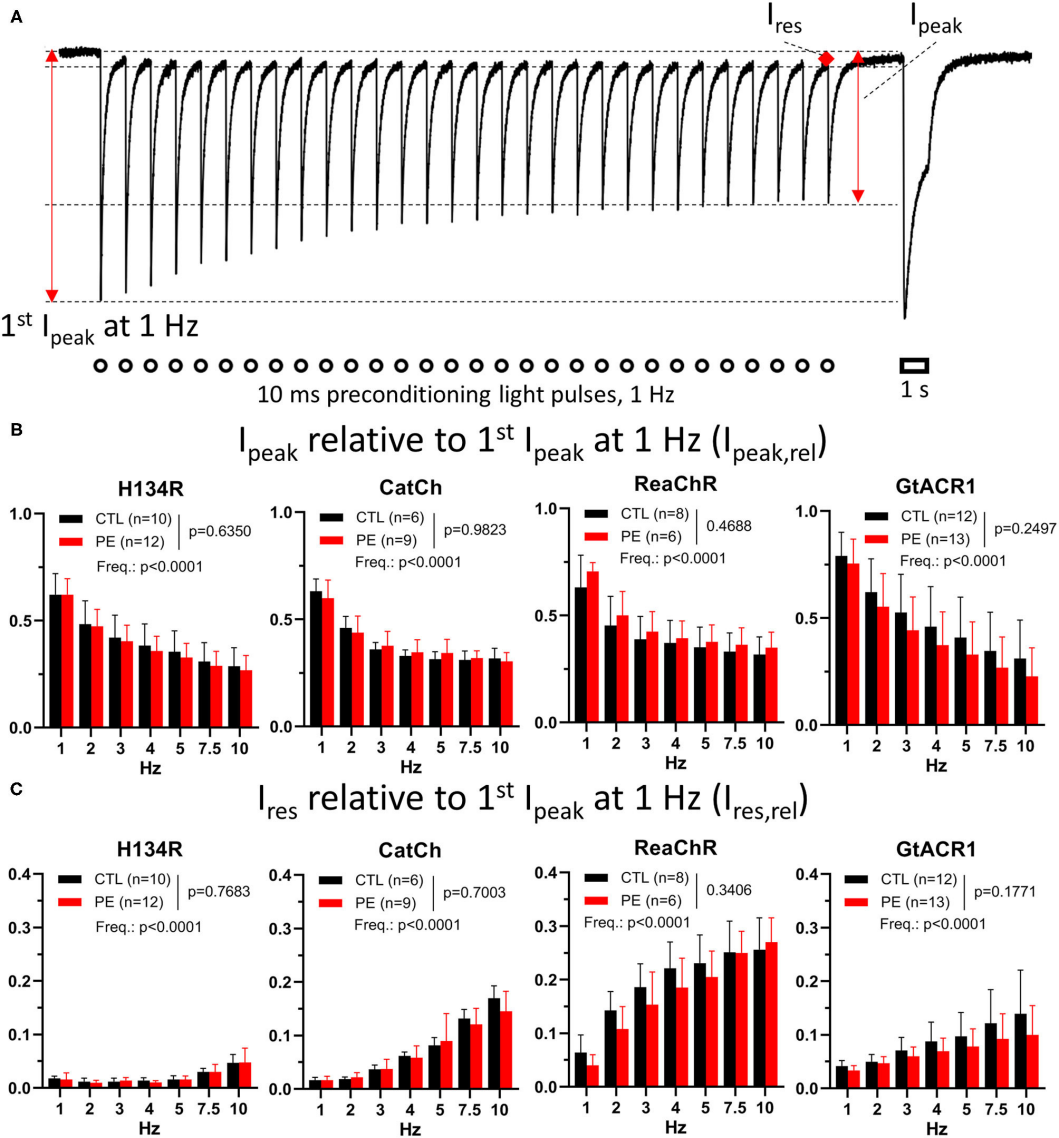
Photocurrent amplitudes during preconditioning by light in CTL and in PE-treated ChR-expressing NRVMs. **(A)** Schematic representation of the quantified parameters and the experimental protocol, overlaid with a single current trace from a ReaChR-expressing CTL NRVM subjected to a 1-Hz preconditioning light pulse train. The preconditioning illumination protocol consisted of brief (i.e., 10-ms) blue (470 nm, for H134R and CatCh), amber (565 nm, for GtACR1), or red (617 nm, for ReaChR) light (1 mW/mm^2^) pulses delivered at a range of frequencies between 1 and 10 Hz for a duration of 30 s. Each preconditioning pulse train was followed by a 1-s light pulse of the corresponding wavelength and identical intensity. The 1-Hz pulse train was applied after a 3-min dark period and was followed without interruption by higher-frequency pulse trains. The quantified current properties included peak current amplitude elicited by the first light pulse of the 1-Hz pulse train (first *I*_peak_ at 1 Hz), peak current (*I*_peak_), as defined by the maximal current amplitude measured at the end of the light pulse train at each frequency and the residual current (*I*_res_), which was measured at the end of the dark period before the last light pulse at each frequency. Open circles and the open bar indicate illumination times. **(B)** Average *I*_peak,rel_ values calculated as *I*_peak_ at the end of the pulse train normalized to the first *I*_peak_ at 1 Hz for each frequency. **(C)** Average *I*_res,rel_ values calculated as *I*_res_ at the end of the pulse train normalized to the first *I*_peak_ at 1 Hz for each cell. Data are presented as mean ± 95% CI for the number of cells (*n*) indicated in each panel. The *p*-values corresponding to the effects of PE treatment vs. CTL conditions and the effect of preconditioning light pulse frequency (Freq.) were calculated by using two-way ANOVA.

The effects of light adaptation on ChR currents were determined, both in PE-treated and in CTL NRVMs. To this end, ChRs were subjected to a preconditioning illumination protocol consisting of trains of 10-ms light pulses (1 mW/mm^2^), delivered at increasing frequencies ranging from 1 to 10 Hz and lasting for 30 s at each frequency (Figure 3A and Supplementary Figure 3). The first preconditioning light pulse train (1 Hz) was preceded by a 3-min dark period allowing complete ChR relaxation and was followed without interruption by the higher-frequency preconditioning illumination protocols. To gain insight into the effects of light adaptation on ChR function, NRVMs were illuminated after each preconditioning pulse train by a light pulse of the same duration, intensity and wavelengths as used to characterize the dark-adapted ChRs. This allowed us to directly compare dark-adapted and light-adapted ChR function and to study the effects of recurrent activation by light in a quantitative manner both in PE-treated and CTL cells.

**Figure 4:**
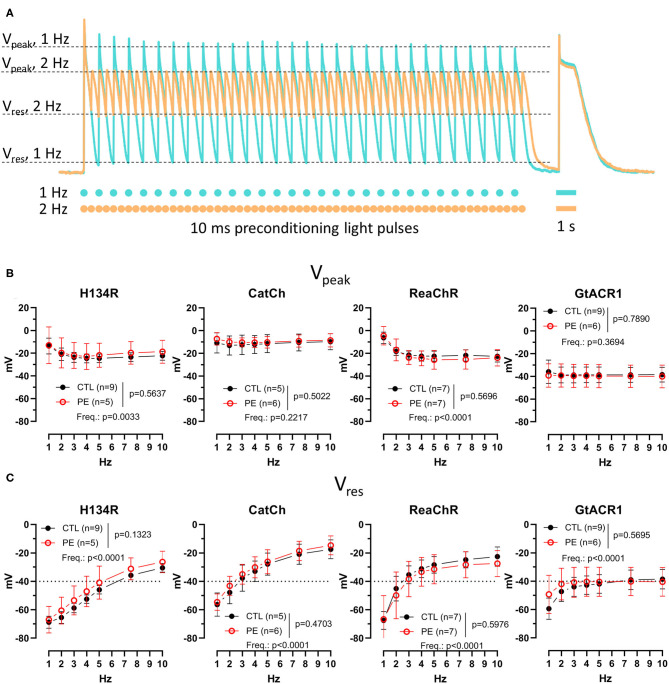
Membrane potential (*V*_m_) response during preconditioning by light in CTL and in PE-treated ChR-expressing NRVMs. **(A)** Schematic representation of the quantified parameters and the experimental protocol, overlaid with representative *V*_m_ recordings for 1-Hz (turquoise) and 2-Hz (apricot) preconditioning pulse trains obtained from an NRVM expressing ReaChR. Dots and bars below the *V*_m_ recording indicate the timing of the 10-ms and 1-s light pulses, respectively. **(B)** Peak *V*_m_ (*V*_peak_) measured at the end of the pulse train. **(C)** Residual *V*_m_ (*V*_res_) measured at the end of the pulse train. Data are presented as mean ± 95% CI for the number of cells (*n*) indicated in each panel. The *p*-values corresponding to the effects of PE treatment vs. CTL conditions and the effect of preconditioning light pulse frequency (Freq.) were calculated by using two-way ANOVA.

**Figure 5:**
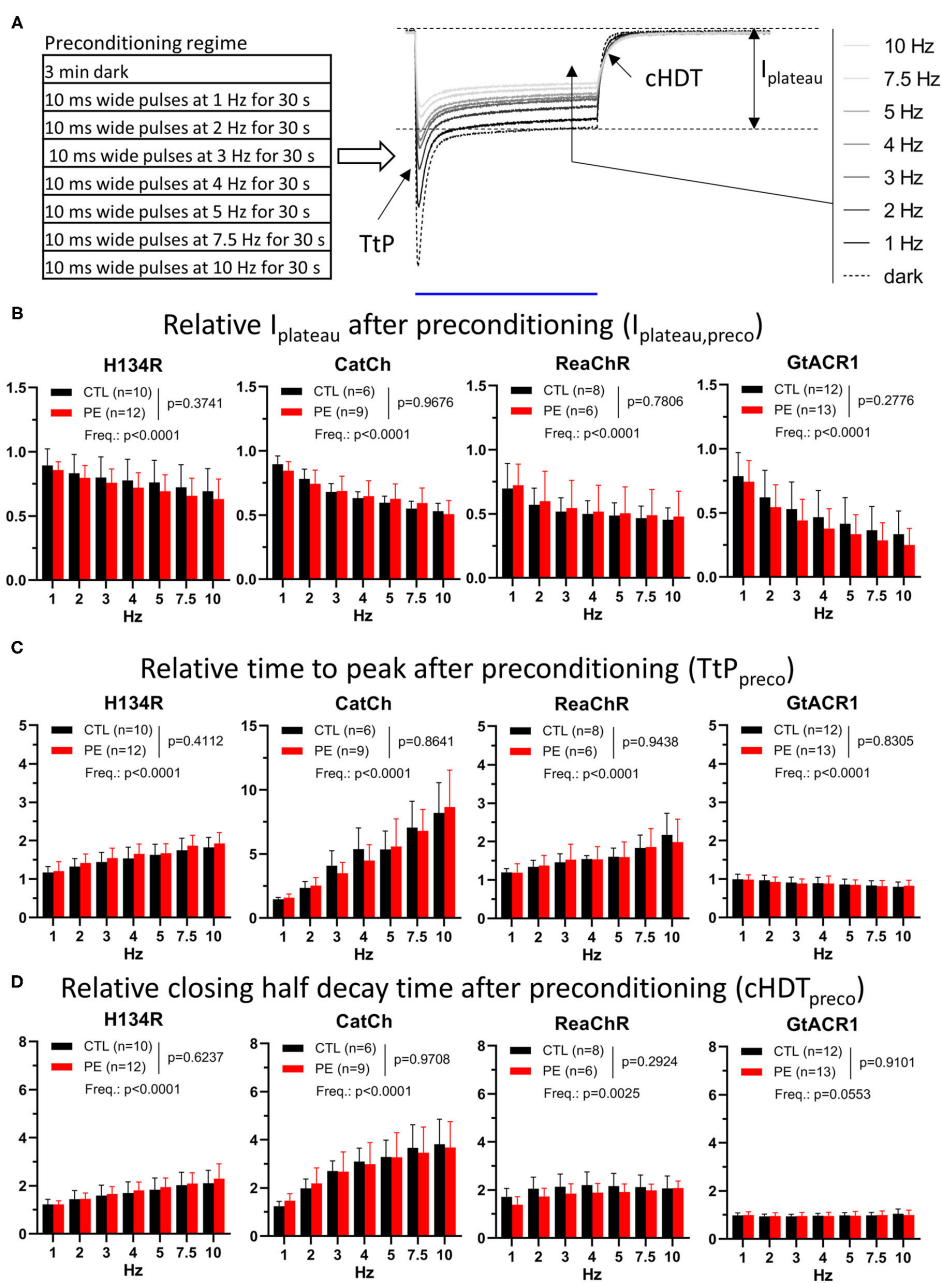
Properties of photocurrents elicited by blue (470 nm, for H134R and CatCh), amber (565 nm, for GtACR1), or red (617 nm, for ReaChR) light pulses of 1 s (1 mW/mm^2^) following preconditioning in CTL and in PE-treated ChR-expressing NRVMs. **(A)** Schematic representation of the experimental protocol (left) and the quantified current parameters together with a representative recording of a series of preconditioned photocurrents from an NRVM expressing the H134R ChR variant (right). The blue line below the current traces represents the 1-s illumination period. **(B)** Preconditioned plateau current amplitudes (*I*_plateau,preco_) relative to *I*_plateau_ observed in the dark-adapted state. **(C)** Preconditioned time to peak (TtP_preco_) relative to TtP observed in the dark-adapted state. **(D)** Preconditioned closing half-decay time (cHDT_preco_) relative to cHDT measured in the dark-adapted state. Each measured value was normalized by the corresponding data point obtained from the experiment carried out in the same cell under dark-adapted conditions (dark) as shown in Figure 1. Data are presented as mean ± 95% CI for the number of cells (*n*) indicated in each panel. The *p*-values corresponding to the effects of PE treatment vs. CTL conditions and the effect of preconditioning light pulse frequency (Freq.) were calculated by using two-way ANOVA.

**Figure 6:**
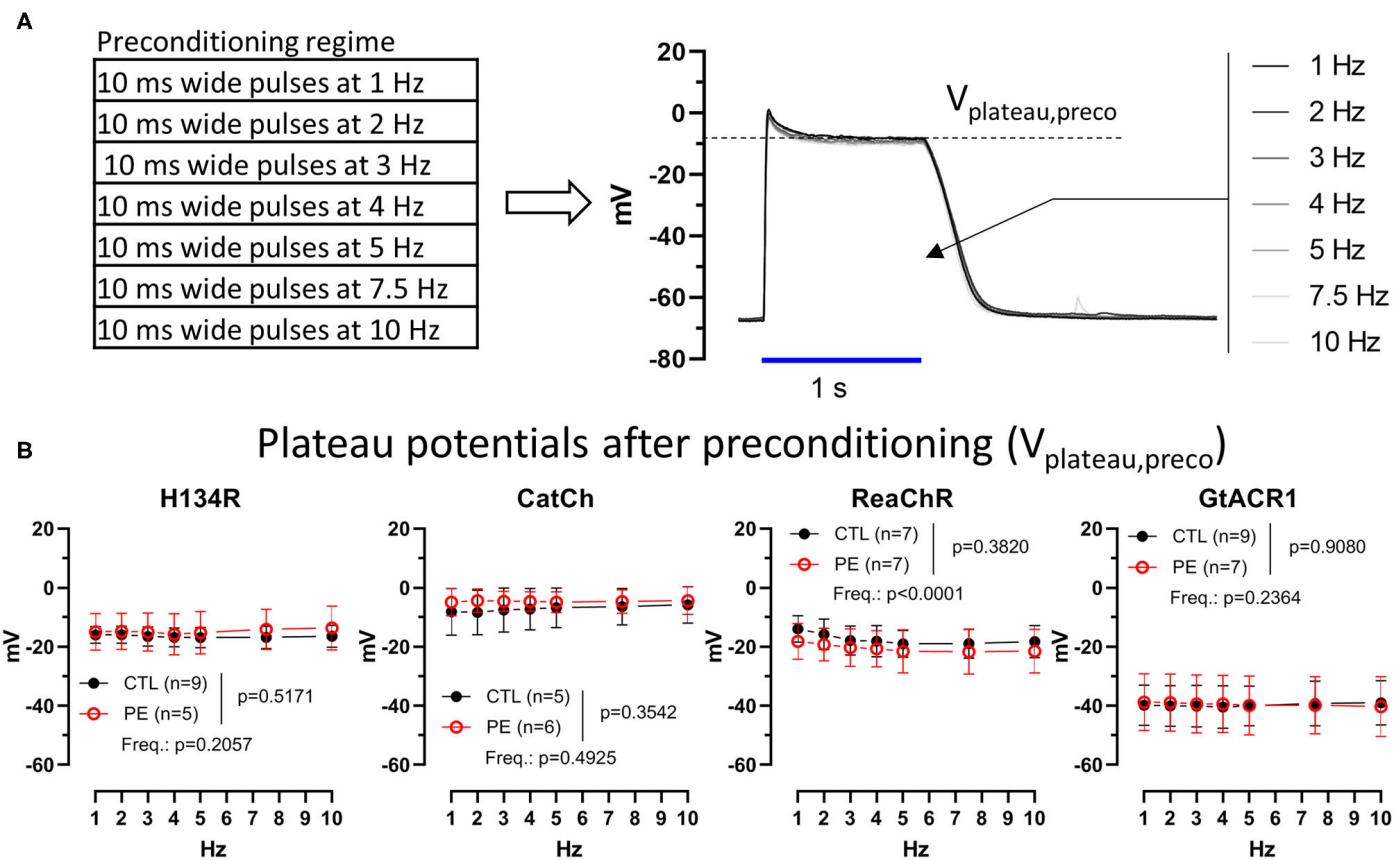
Membrane potential (*V*_m_) response to a 1-s blue (470 nm, for H134R and CatCh), amber (565 nm, for GtACR1), or red (617 nm, for ReaChR) light pulse (1 mW/mm^2^) following preconditioning in CTL and PE-treated ChR-expressing NRVMs. **(A)** Schematic representation of the experimental protocol (left) and a representative example of a series of *V*_m_ traces recorded from an NRVM expressing H134R following preconditioning by different optical pacing frequencies (right). **(B)** Plateau potentials as the function of preconditioning pulse train frequency. Data are presented as mean ± 95% CI for the number of cells (*n*) indicated in each panel. The *p*-values corresponding to the effect of PE treatment vs. CTL conditions and the effect of preconditioning light pulse frequency (Freq.) were calculated by using two-way ANOVA.

The data obtained by this programmed illumination regime are presented as follows. ChR current behavior and the concomitant *V*_m_ response during the preconditioning pulse train are presented further in the “Membrane potential (*V*_m_) response to preconditioning illumination protocol” section and in Figures 3, 4, whereas ChR current properties and changes in *V*_m_ elicited by the prolonged illumination following each preconditioning pulse train are analyzed in the “Effects of preconditioning by light on photocurrents,” “Membrane potential (*V*_m_) response to preconditioned photocurrents” sections and in Figures 5, 6, respectively.

First, ChR current behavior under preconditioning illumination protocols was assessed. Peak currents were measured at the end of the pulse train of each frequency and were normalized to the peak current triggered by the first light pulse of the 1-Hz pulse train representing the dark-adapted state, yielding relative peak currents (*I*_peak,rel_, Figure 3A). In general, average *I*_peak,rel_ values decreased with an increase in the frequency of the preconditioning light pulse train (Figure 3B). At the end of the first train of light pulses (1 Hz train), *I*_peak_ was ~35% lower for H134R, CatCh, and ReaChR than at its beginning. Of all ChR variants, *I*_peak_ was most stable (*p* < 0.05) under the 1-Hz preconditioning illumination protocol for GtACR1, showing only ~25% decrease compared with the dark-adapted state (Figure 3B). Pathological hypertrophy, as induced by PE treatment, had no effect on average *I*_peak,rel_ values, whereas the effect of pulse train rate was highly significant for each ChR variant (Figure 3B). Linear regression of the data presented in Figure 3B confirmed the existence of a negative correlation between *I*_peak,rel_ and preconditioning frequency by yielding significantly non-zero negative slopes (*p* < 0.0001) for all ChR variants, with the steepest negative slope being calculated for GtACR1 (*p* < 0.0001), indicating a stronger use-dependence of the GtACR1 *I*_peak_ compared with other variants (Supplementary Figure 7A).

Residual currents (*I*_res_) were measured 1 ms before the application of the last light pulse of each train and were normalized to the first peak current of the 1-Hz pulse train, yielding *I*_res,rel_ (Figure 3C). Average *I*_res,rel_ values were not different in PE-treated compared with CTL cells (*p* > 0.05), while the effect of pulse train frequency was highly significant (*p* < 0.0001, Figure 3C). *I*_res,rel_ observed at the end of the 1-Hz pulse train was smallest for H134R (*p* < 0.05) and largest for ReaChR (*p* < 0.05). Linear regression revealed that *I*_res,rel_ increased with increasing frequency of the preconditioning pulse train, i.e., for each ChR variant, a significantly non-zero positive slope (*p* < 0.0001) was obtained. Slopes of trend lines were smallest for H134R (*p* < 0.05), significantly larger for GtACR1 (*p* < 0.05), and largest for CatCh and ReaChR (*p* < 0.05, Figure 3C and Supplementary Figure 7B). Notably, the variants displaying the highest frequency-dependence of *I*_res_ (i.e., CatCh and ReaChR) also showed the slowest closing kinetics in the dark-adapted state (cHDT, Figure 1J), indicating the existence of a correlation between these two functional parameters.

### Membrane Potential (*V*_m_) Response to Preconditioning Illumination Protocol

The *V*_m_ response during the preconditioning illumination protocol was characterized by two distinct indicative *V*_m_ values for each pulse train. *V*_peak_ was defined as the most positive *V*_m_ value observed during the last duty cycle of each pulse train, whereas *V*_res_, to the analogy of *I*_res_, was measured as the *V*_m_ value within 1 ms preceding the onset of the last light pulse of each pulse train (Figure 4A and Supplementary Figure 4). Neither *V*_peak_ nor *V*_res_ values were different in PE-treated NRVMs compared with CTL cells (*p* > 0.05, Figures 4B,C). Pulse train frequency had a significant effect on *V*_peak_ for H134R and ReaChR (*p* < 0.05), but not for CatCh and GtACR1 (Figure 4B). In cells expressing H134R or ReaChR, *V*_peak_ values decreased with increasing preconditioning pulse train frequency until 3 Hz, but stabilized between −20 and −25 mV at higher frequencies of the light pulses. Average *V*_peak_ values were within the range of −10 ± 2 mV for CatCh and −38 ± 2 mV for GtACR1, irrespective to the preconditioning pulse train frequency (Figure 4B). Similar to *V*_peak_, average *V*_res_ values remained unaffected by PE treatment, but were highly dependent on pulse train frequency (*p* < 0.0001, Figure 4C). *V*_res_ increased with increasing preconditioning pulse rate for all ChR variants. In cells expressing the H134R variant, *V*_res_ remained < −40 mV at preconditioning pulse train frequencies of up to 5 Hz. However, for CatCh and ReaChR, *V*_res_ values already became >−40 mV at an optical pacing frequency of 3 Hz. *V*_res_ for GtACR1 approached −40 mV when the pulse train frequency was increased from 1 to 3 Hz and remained relatively stable later (Figure 4C).

### Effects of Preconditioning by Light on Photocurrents

As mentioned above, in order to elicit (partially) light-adapted photocurrents in a standardized way, each preconditioning light pulse train was followed by an additional illumination period of 1 s (Figure 5A and Supplementary Figure 5). The preconditioned photocurrents captured in this way were used to assess the effects of light adaptation on ChR function both in PE-treated and in CTL NRVMs. Properties of the preconditioned photocurrents were determined and normalized to the same parameters obtained for the dark-adapted ChR state (Figures 1, 2) for each cell, yielding relative parameters, herein labeled with the “preco” suffix.

Strikingly, preconditioning induced a decrease in *I*_peak_, *I*_plateau_, and TC. However, each current property responded differently to preconditioning with relative changes characteristic for each ChR variant. *I*_peak_ decreased significantly (*p* < 0.05) for all four ChR variants after 1-Hz preconditioning, with GtACR1 showing the smallest (*p* < 0.05) decrease (CTL: 17%, PE: 24%) (Supplementary Figure 6A and Supplementary Table 1). Delivery of preconditioning pulse trains of higher w *I*_peak,preco_. As a consequence, plotting *I*_peak,preco_ values against preconditioning pulse frequency yielded lines with non-zero (*p* < 0.0001) negative slopes for all four ChR variants, indicating strong use-dependence of *I*_peak,preco_ (Supplementary Figure 7C and Supplementary Table 1). Similar to peak currents, plateau currents were also affected by light adaptation. *I*_plateau_ showed a significant (*p* < 0.05) decrease of >10% in response to 1-Hz preconditioning relative to dark-adapted state for all four ChR variants and decreased further with increasing preconditioning intensity (*p* < 0.0001) (Figure 5B, Supplementary Figure 7D and Supplementary Table 1). Interestingly, the plateau current generated by ReaChR appeared to be the least resistant to repetitive use among the cation ChR variants, since it showed the smallest average *I*_plateau,preco_ values (CTL: 30%, PE: 28%) (*p* < 0.05) after 1-Hz preconditioning. In accord with the use-dependent decrease in *I*_peak,preco_ and *I*_plateau,preco_ amplitudes, all ChR currents carried a lower amount of charge during 1-s illumination following 1-Hz preconditioning compared with dark-adapted currents (*p* < 0.05) with ReaChR, showing the largest average decrease (CTL: 33%, PE: 35%) compared with any other cation ChR variant (*p* < 0.05) (Supplementary Figures 6B, 7E and Supplementary Table 1). It is also noteworthy that *I*_peak_−*I*_plateau_ ratios of the cation ChRs decreased (*p* < 0.05) following 1-Hz preconditioning and decreased further with increased preconditioning frequencies (Supplementary Figures 6C, 7F and Supplementary Table 1). 1-Hz preconditioning had no significant effect on GtACR1 *I*_peak_−*I*_plateau_ ratios (*p* > 0.05), but higher pulse rates caused increasing trends with non-zero (*p* < 0.0001) positive slopes (Supplementary Figures 6C, 7F and Supplementary Table 1). Therefore, our data show that ChRs generate lower current amplitudes, thus conducting less charge in the light-adapted state than in the dark-adapted state. The preconditioning-induced changes in any of the assessed quantitative current properties, however, were not different between CTL and PE-treated cells (*p* > 0.05) (Figure 5B, Supplementary Figures 6A–C, and Supplementary Table 1).

Channelrhodopsin current amplitudes are not the sole determinants of the efficacy of optogenetic maneuvers. The kinetic properties of ChRs, such as activation and deactivation times, may critically limit the temporal resolution of optogenetic interventions, yet their stability under conditions of repetitive use and in the setting of cardiac disease is unknown. Therefore, we compared the kinetic properties of preconditioned ChR currents with those measured in the dark-adapted state (Figure 5A and Supplementary Figure 5). Activation kinetics of all cation ChR variants, represented by TtP_preco_, decelerated (*p* < 0.05) following preconditioning, as indicated by average TtP_preco_ values >1 (Figure 5C and Supplementary Table 2). CatCh decelerated the most, showing a >45% increase of average TtP following 1-Hz preconditioning (CTL: 46%, PE: 59%). These changes were exaggerated following more intense preconditioning for all cation ChRs, as all average TtP_preco_ values showed trends having non-zero (*p* < 0.0001) positive slopes, with the highest (*p* < 0.05) slope for CatCh (Figure 5C, Supplementary Figure 7G and Supplementary Table 2). Surprisingly, the kinetics of GtACR1 current activation did not change following 1-Hz preconditioning (*p* > 0.05), but GtACR1 TtP_preco_ decreased slightly with higher preconditioning pulse train rates showing a trend with a significantly non-zero (*p* < 0.0001) negative slope (Figure 5C, Supplementary Figure 7G and Supplementary Table 2). Use-dependent deceleration of channel activation was accompanied by slowing of channel closure in case of the cation ChR variants (Figure 5D and Supplementary Table 2). cHDT values following 1-Hz preconditioning were increased by >20% for all cation ChRs relative to dark-adapted cHDT values. Higher preconditioning pulse rates induced further deceleration of ion channel closure, as indicated by non-zero (*p* < 0.05), positive average cHDT_preco_ trends with steeper slopes for H134R compared with ReaChR (*p* < 0.05) and for CatCh compared with H134R (*p* < 0.05) (Figure 5D and Supplementary Figure 7H). Interestingly, GtACR1 cHDT was affected neither by 1-Hz preconditioning (CTL: 2%, PE: 1%, *p* > 0.05) nor by higher optical pacing frequencies (*p* = 0.0553, Figure 5D, Supplementary Figure 7H and Supplementary Table 2). Inactivation kinetics, as characterized by iHDT, decelerated >10% for H134R and >2-fold for CatCh following 1-Hz preconditioning pulses compared with dark-adapted iHDT values and slowed even more with more intense preconditioning (Supplementary Figure 6D and Supplementary Table 2). In this comparison, CatCh showed the highest (*p* < 0.05) positive slope of average iHDT_preco_ values among all ChR variants. The inactivation kinetics of ReaChR and GtACR1 were not affected by 1-Hz preconditioning, but while the former showed a trend of deceleration, the latter showed acceleration with higher pulse rates (Supplementary Figures 6D, 7I and Supplementary Table 2). Importantly, the effects of light adaptation on ChR kinetics did not differ (*p* > 0.05) between CTL and PE-treated NRVMs (Figures 5C,D and Supplementary Figures 6C,D, 7G–I).

In summary, these data show strong use-dependence of important characteristics of ChR currents, including nearly all quantitative and kinetic properties of the photocurrents produced by the four ChR variants, while these parameters remained unchanged under conditions of cellular hypertrophy. Such use-dependence may result in decreased current amplitudes reducing ChR performance, as well as altered temporal responses to repetitive light activation and thus has to be taken into account during the development of any optogenetic application seeking to employ ChR variants. These findings predict that the efficacy of optogenetic applications might become limited when repeated ChR activation is required.

### Membrane Potential (*V*_m_) Response to Preconditioned Photocurrents

The *V*_m_ response to light-adapted ChR activation was assessed by quantifying plateau potentials following ChR preconditioning by light (*V*_plateau,preco_) both in CTL and in PE-treated NRVMs under the same illumination regime that was used to compare dark-adapted and (partially) light-adapted ChR current properties (Figure 6 and Supplementary Figure 8). We found that average *V*_plateau_ values did not differ significantly (*p* > 0.05) following preconditioning with a 1-Hz pulse train compared with dark-adapted *V*_plateau_ values for any of the ChR variants (Figure 6B). Preconditioning pulse rate had no effect on average *V*_plateu_ in case of H134R, CatCh, and GtACR1 (*p* > 0.05). ReaChR *V*_plateau_ decreased slightly with increasing preconditioning pulse frequency; however, linear regression failed to detect a significantly non-zero slope (*p* > 0.05). PE stimulation had no effect on average *V*_plateau_ values (*p* > 0.05) (Figure 6B).

## Discussion

In this study, we compared the functioning of different ChR variants in cardiomyocytes under normal and pathological conditions when challenged by various illumination protocols. The results reveal that ChR function remains largely unaltered in a cellular model of pathological hypertrophy but shows strong, variant-specific use-dependence. This use-dependent nature of ChR function should not only be considered in the selection of a certain ChR variant for a given purpose but also in the interpretation of the experimental findings.

The rationale for this study was 3-fold. First, ChR variants are the most frequently used optogenetic tools in cardiac research. There is a rich and diverse repertoire of ChR-based tools with unique biophysical properties, including ion selectivity, conductivity, kinetic properties, and light absorption spectra. The combination of these properties is crucial for the successful application of ChRs for both basic and translational research purposes. Nevertheless, studies carried out under rigorously standardized conditions are scarce and have thus far been conducted in permanent (non-cardiac) cell lines, *Xenopus laevis* oocytes, and neurons (Nagel et al., 2003; Lin, 2011; Mattis et al., 2011). Considering that the time course and changes in the magnitude of *V*_m_ caused by ChR currents critically depend on the properties of the host cell membrane, the characterization of ChR behavior in cardiomyocytes is vital for optimal application of optogenetics in cardiac research. Therefore, we used standardized experimental conditions to perform a reliable, side-by-side comparison of important ChR variant properties that cardiac applications crucially rely on, such as *I*_peak_ and TtP for cardiac pacing and *I*_plateau_ and *V*_plateau_ for cardiac arrhythmia termination.

Second, considering that both basic research and translational studies often target the diseased heart, testing ChR function in the setting of cardiac disease is pivotal. Pathological cardiac hypertrophy is a maladaptive response to increased hemodynamic stress and is associated with a large variety of inherited and acquired cardiac diseases (Frey and Olson, 2003; Bernardo et al., 2010; Nakamura and Sadoshima, 2018). Such hypertrophy involves structural and electrical remodeling at the cellular level, thereby potentially limiting ChR performance. NRVM cultures are the most used and one of the best characterized cellular cardiac electrophysiological models (Askar et al., 2011; Bingen et al., 2013, 2014; Feola et al., 2016; Majumder et al., 2016, 2018). In response to chronic adrenergic stimulus, NRVMs undergo marked structural and electrical remodeling and show characteristic features of pathological cardiac hypertrophy, such as cell enlargement, APD prolongation, an increase in total protein content, and reactivation of “fetal” genes including the ANP-encoding *NPPA* gene (Eble et al., 1998; Gaughan et al., 1998; Bernardo et al., 2010; Askar et al., 2013; Neshati et al., 2020). These features make NRVM cultures a very suitable and relevant cardiomyocyte-based model for the functional characterization of optogenetic tools both under physiological conditions and in the setting of pathological hypertrophy.

Third, the use of optogenetic tools to control the *V*_m_ of cardiomyocytes often involves repeated activation of ChRs by using the application of multiple consecutive light pulses (Bingen et al., 2014; Park et al., 2014; Govorunova et al., 2016; Feola et al., 2017; Majumder et al., 2018). Such repetitive use is intrinsic to the application of ChRs for optogenetic pacing, but also ChR-based termination of arrhythmias might rely on more than one light pulse. In fact, termination of cardiac arrhythmias by light might fail at the first attempt, in which a second light flash or more light pulses are required (Bruegmann et al., 2018; Nyns et al., 2019). Importantly, if cardiac optogenetics would enter the phase of clinical exploration, reaching optimal efficacy and safety would be of paramount importance. This makes the requirement for optimal ChR performance during repeated illumination even more pivotal, especially considering other challenges, like those concerning effective and safe delivery of both the transgene (like ChR) and light (e.g., *via* a biocompatible LED device). Previous optogenetic studies in the field of neurology revealed that ChR currents are prone to inactivation, which means that *I*_peak_ decreases during consecutive illumination pulses and that a dark period lasting for several seconds is required to regain its original amplitude (Boyden et al., 2005; Lin et al., 2009; Schneider et al., 2015), the insight that we have currently extended to the field of cardiology. This aspect of photocurrent inactivation, combined with the potentially challenging features of transgene expression and light delivery, might compromise the efficacy of optogenetic applications in the translational and clinical setting. Therefore, in this study, we investigated and compared photocurrent properties under conditions that allowed the complete dark adaptation of ChRs and graded light adaptation afterwards by exposing ChRs to illumination regimens of increasing intensity.

In terms of how our results could help the selection of the best-suited ChR variant for a given application, the dark-adapted current properties provide an initial selection guide. For example, the H134R variant produces the lowest current amplitudes, while it is also the fastest to activate. The H134R variant, therefore, might be an ideal choice for applications requiring fast activation, provided that sufficiently high ChR gene expression and irradiation levels can be achieved. Our data show that CatCh generates the largest current amplitudes among the cation ChR variants investigated in this study. However, it is worth noting that CatCh activates slower than H134R, potentially limiting its applicability when fast activation is required. Since visible light of longer wavelengths penetrates biological tissues better than visible light of shorter wavelengths, the use of red-shifted variants such as ReaChR is considered to be beneficial in *in vivo* experiments. Although ReaChR delivers photocurrents with similar amplitudes as H134R, the remarkably slower activation and closing kinetics of ReaChR potentially resulting in end-diastolic depolarization may limit its usefulness for experiments requiring high optical pacing rates. Finally, GtACR1 seems more suited for optical pacing than for arrhythmia termination because of its relatively modest depolarizing effect on NRVMs.

In contrast, the properties of dark-adapted ChR currents may not be the only aspect of ChR function one should consider in search of the ideal ChR variant. Our data show that all ChR variants included in this study generated reduced current amplitudes and displayed characteristically different kinetic properties in the light-adapted state compared with the dark-adapted state and some of these changes translated into differential cell membrane responses as well. Most importantly, in accord with the well-known phenomenon of ChR current inactivation (Lin, 2011; Schneider et al., 2015), preconditioning decreased average *I*_peak_ for all four ChR variants. The use-dependent *I*_peak_ decrease was accompanied by a reduction of *I*_plateau_. *I*_plateau_ is an important parameter of ChR function when employed for arrhythmia termination since sustained membrane depolarization and the resulting conduction block critically depend on the amount of current that remains relatively stable during long-lasting (i.e., 100-ms to 1-s) illumination. Under optimal conditions, where ChR expression levels are not limited, the use-dependent *I*_plateau_ reduction may not translate into a diminished ability to maintain membrane depolarization. This scenario is exemplified by the experimental settings employed in this study, as average *V*_plateau_ values remained independent of the amount of preconditioning in most experimental groups. However, one may speculate that under limiting conditions, where the ChR expression levels are suboptimal, the use-dependent reduction of *I*_plateau_ might lead to less depolarized *V*_plateau_ values.

The use-dependent reduction of *I*_plateau_ amplitudes has not been documented earlier but may well be in line with the most recent ChR photocycle model (Kuhne et al., 2019). According to this model, ChRs can occupy two parallel photocycles, the “anti-cycle,” which involves an early and a late open state (O_1−early_ and O_1−late_, respectively) with higher and lower conductivity, respectively, and the “syn-cycle,” which enrolls a third open O_2_ state with even lower conductivity (Kuhne et al., 2019). Branching between the anti- and syn-cycles takes place in the dark-adapted, closed state (C_1_) under illumination, while relaxation from the syn- to the anti-cycle is possible *via* two routes. Light-induced branching is characterized by a fast time constant (<1 ns), whereas relaxation from the syn- to the anti-cycle *via* any of the two possible routes is slower by several orders of magnitude (250 ms and 40 s). Therefore, it is possible that ChRs activated by a single light pulse following sufficiently long dark adaptation will reside in the anti-cycle predominantly. However, the relative occupancy of the two photocycles may shift toward the syn-cycle during recurrent use, thereby favoring occupancy of the less conductive O_2_ state.

In this study, we showed that repetitive activation causes use-dependent reduction of ChR current amplitudes and altered ChR kinetics. Such use-dependence might limit the efficacy of optogenetic interventions under suboptimal conditions and hence requires careful evaluation depending on the specific application. In addition, efforts directed at the engineering of new ChR variants with tailored properties for particular purposes should take this ChR use-dependency into consideration to maximize experimental outcomes (Bedbrook et al., 2019).

Activation of ChRs leads to robust depolarization of the cell membrane, which under ideal conditions, can be maintained for a virtually unlimited time. During this time, large amounts of sodium and calcium ions enter the cell quickly exceeding physiological intracellular ion concentrations when the illumination is maintained. These conditions may represent stress to which cardiomyocytes have never been exposed and will likely result in as yet unexplored cellular responses. Future translational studies thus should be focused not only on the efficacy of optogenetic interventions but also on the possible adverse side effects.

Importantly, despite the characteristic structural and electrical remodeling present in PE-treated NRVMs, *V*_m_ changes induced by ChR activation and the properties of the underlying ChR currents did not differ between CTL and PE-treated cells. These findings support the use of cardiac optogenetics to modulate the electrical activity of cardiomyocytes both under normal conditions and in the setting of cardiac disease for fundamental and translational research purposes and also eventually for clinical applications.

## Data Availability Statement

The original contributions presented in the study are included in the article/Supplementary Material, further inquiries can be directed to the corresponding author/s.

## Ethics Statement

The animal study was reviewed and approved by the Animal Experiments Committee of the Leiden University Medical Center.

## Author Contributions

BÖ conceptualized the research, designed and carried out experiments, analyzed and interpreted the data, and wrote the manuscript. AT interpreted the data and reviewed the manuscript. TDC contributed to software development and reviewed the manuscript. CIB, SOD, and JZ helped with molecular cloning, cell culture and immunocytochemical staining, and reviewed the manuscript. DLY interpreted the data and critically evaluated and reviewed the manuscript. AAFdV and DAP conceptualized the research, designed experiments, reviewed and edited the manuscript. All authors approved the submitted version.

## Conflict of Interest

The authors declare that the research was conducted in the absence of any commercial or financial relationships that could be construed as a potential conflict of interest.

## Publisher's Note

All claims expressed in this article are solely those of the authors and do not necessarily represent those of their affiliated organizations, or those of the publisher, the editors and the reviewers. Any product that may be evaluated in this article, or claim that may be made by its manufacturer, is not guaranteed or endorsed by the publisher.

## Footnotes

^1^Harden, S.W. (2020). pyABF 2.2.3. https://pypi.org/project/pyabf/
