# Supplementary material for: The Effects of Repetitive Use and Pathological Remodeling on Channelrhodopsin Function in Cardiomyocytes
Source: Front Physiol. 2021 Aug 23;12:710020. doi: 10.3389/fphys.2021.710020 (PMC8448166; doi:10.3389/fphys.2021.710020)
Supplement: Supplementary file 1 [file Data_Sheet_1.PDF]

Supplementary Material

1 Supplementary Data

1.1 Phenylephrine (PE)-induced hypertrophic remodeling in neonatal rat ventricular myocytes (NRVMs)

Structural and electrical remodeling was evident in PE-stimulated NRVMs as early as 48 hours after PE administration (**Figure S1**). Immunocytochemical labeling for α-actinin and atrial natriuretic peptide (ANP) was used to assess cell area and to confirm the induction of pathological hypertrophy (**Figure S1A**) (Askar et al., 2011). The average cell area was enlarged (10.4%, p=0.0241, **Figure S1B**) and ANP expression showed a marked increase (39.3%, p<0.0001, **Figure S1C**) in PE-treated NRVMs compared to CTL cells. Moreover, the average total cellular protein content was increased by 80% and 56% in PE-treated compared to CTL NRVM cultures at 48 (Day 5) and 72 (Day 6) hours following PE administration, respectively, indicating a rapid and robust increase of protein synthesis in response to PE exposure (p<0.0001, **Figure S1D**). Furthermore, PE-exposed NRVMs displayed markedly prolonged APs (p=0.013, **Figure S1E, S1F**) in comparison with CTL cells. These findings are consistent with pathological cardiac structural and electrical remodeling confirming that PE treatment rendered NRVMs pathologically hypertrophic.

Supplementary Figure S1

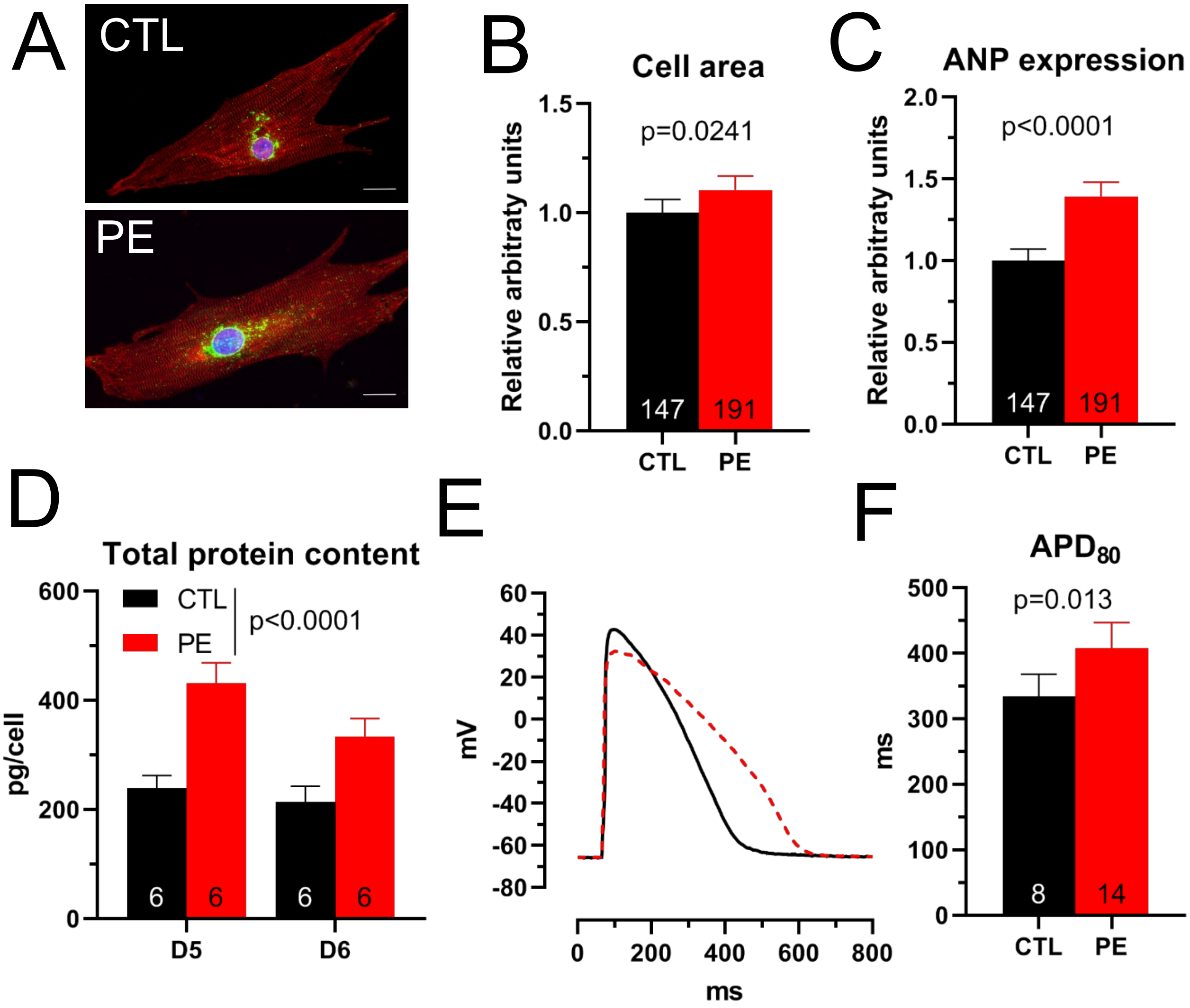

Supplementary Figure S1. Structural and electrical remodeling in control (CTL) and PE-treated (PE) NRVMs. **A**: Photomicrographs of control (CTL) and PE-treated (PE) NRVMs following immunofluorescent labeling of α-actinin (red), ANP (green) and DNA (blue). Scale bars indicate 20 µm. **B**: Quantification of cell area in CTL and PE-treated NRVMs. **C**: Quantification of ANP expression in CTL and PE-treated NRVMs. **D**: Total cellular protein content of CTL and PE-treated cells on Day 5 (D5) and Day 6 (D6) of culture. **E**: Typical electrically triggered action potentials of CTL (black) and PE-treated (red) NRVMs. **F**: APD_{80} values observed in CTL and

in PE-treated NRVMs. Numerical data are presented as mean ± 95% confidence interval. The number of cells (**B**, **C**, **F**) or cultures (**D**) that were analyzed are indicated in the bar graphs.

Supplementary Figure S2

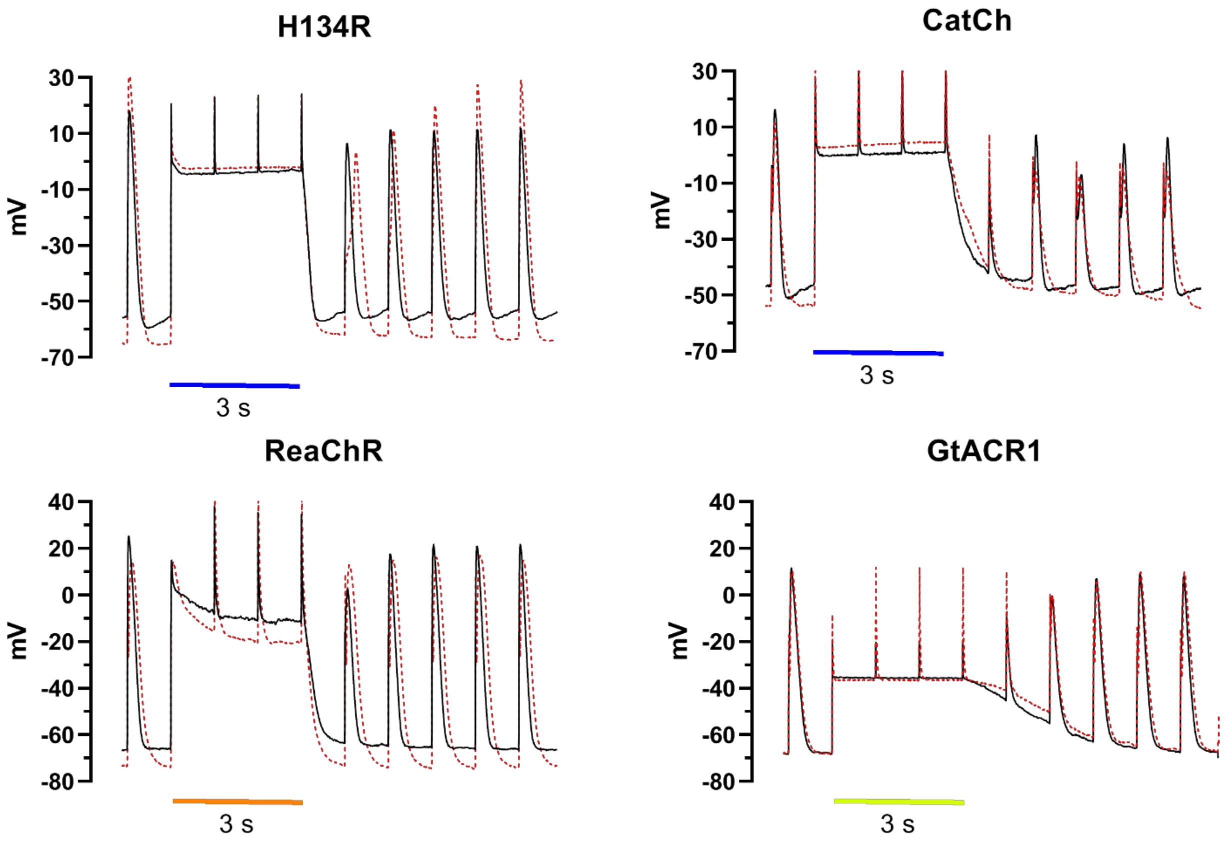

Supplementary Figure S2. Representative membrane potential recordings of electrically triggered APs during illumination in CTL (black) and in PE-treated (red) ChR-expressing NRVMs. NRVMs were illuminated for 3 s using blue (470 nm, for H134R and CatCh), amber (565 nm, for GtACR1) or red (617 nm, for ReaChR) light (1 mW/mm^2) during continuous electrical pacing at 1 Hz.

Supplementary Figure S3

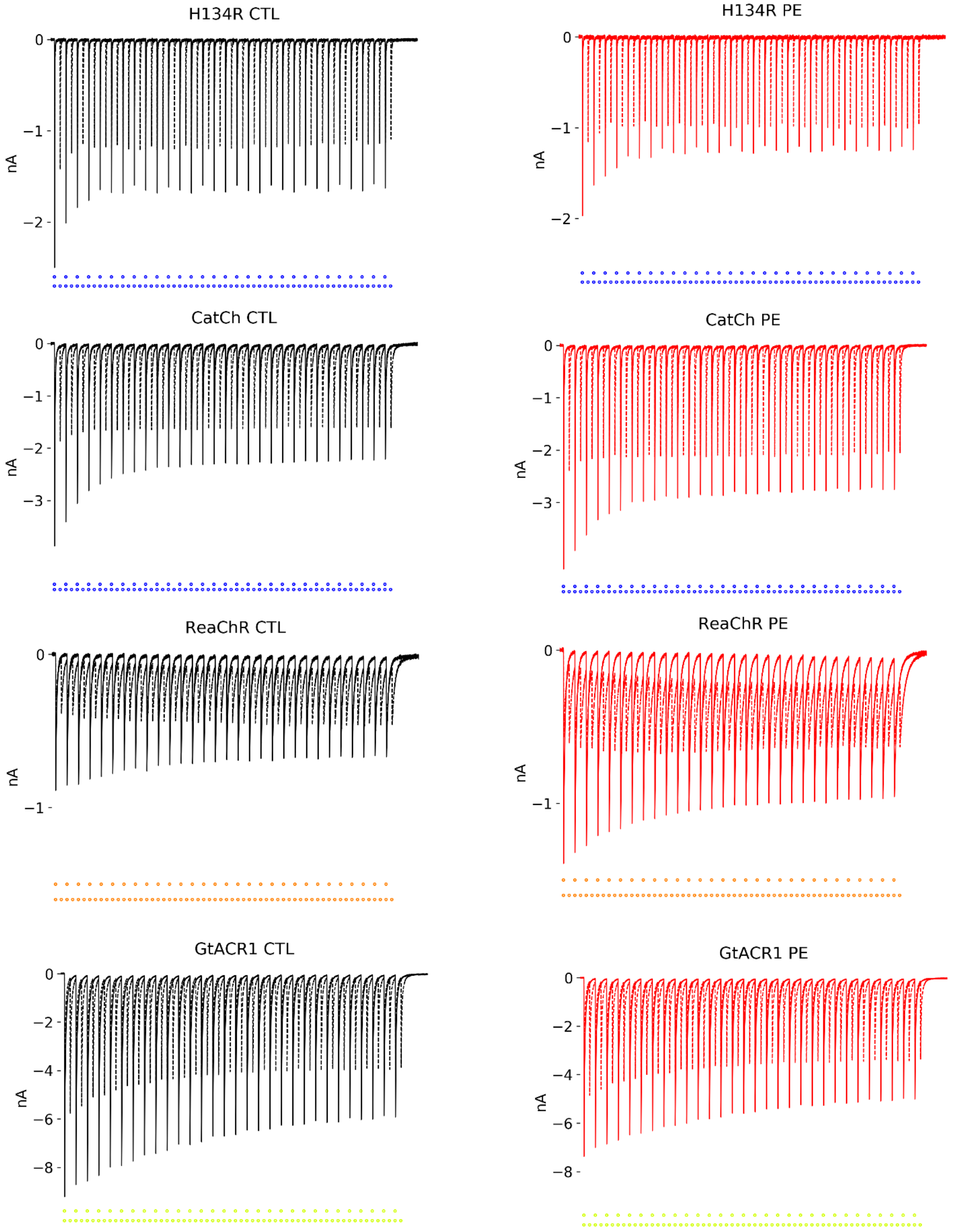

Supplementary Figure S3. Representative recordings of photocurrents elicited by the preconditioning illumination protocol using blue (470 nm, for H134R and CatCh), amber (565 nm, for GtACR1) or red (617 nm, for ReaChR) light (1 mW/mm^2) in CTL (black) and in PE-treated (red) ChR-expressing NRVMs. Only the photocurrents elicited by the light pulse trains of 1 and 2 Hz are shown overlaid for clarity.

Supplementary Figure S4

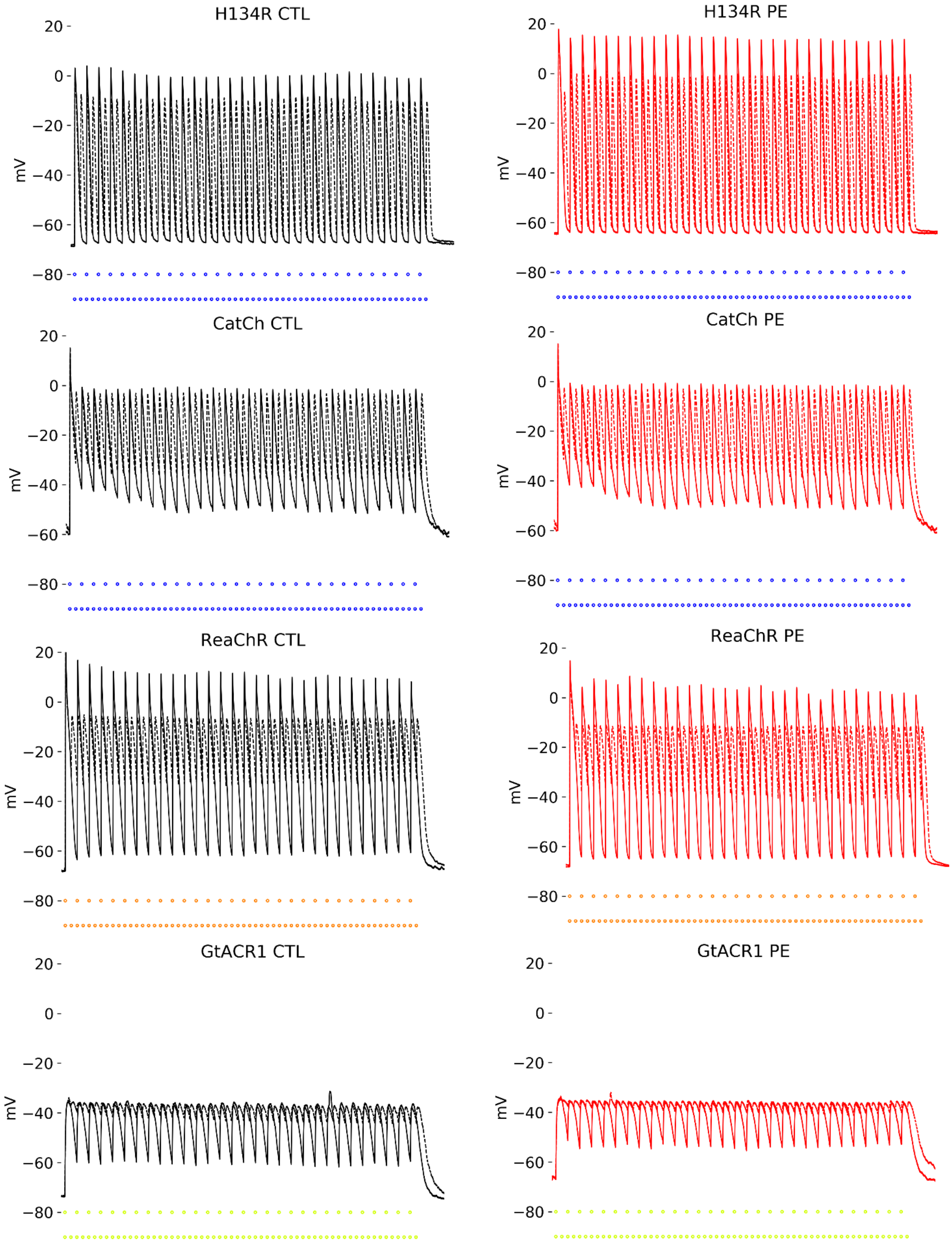

Supplementary Figure S4. Representative membrane potential recordings elicited by the preconditioning illumination protocol using blue (470 nm, for H134R and CatCh), amber (565 nm, for GtACR1) or red (617 nm, for ReaChR) light (1 mW/mm^2) in CTL (black) and in PE-treated (red) ChR-expressing NRVMs. Only the voltage changes elicited by the light pulse trains of 1 and 2 Hz are shown overlaid for clarity.

Supplementary Figure S5

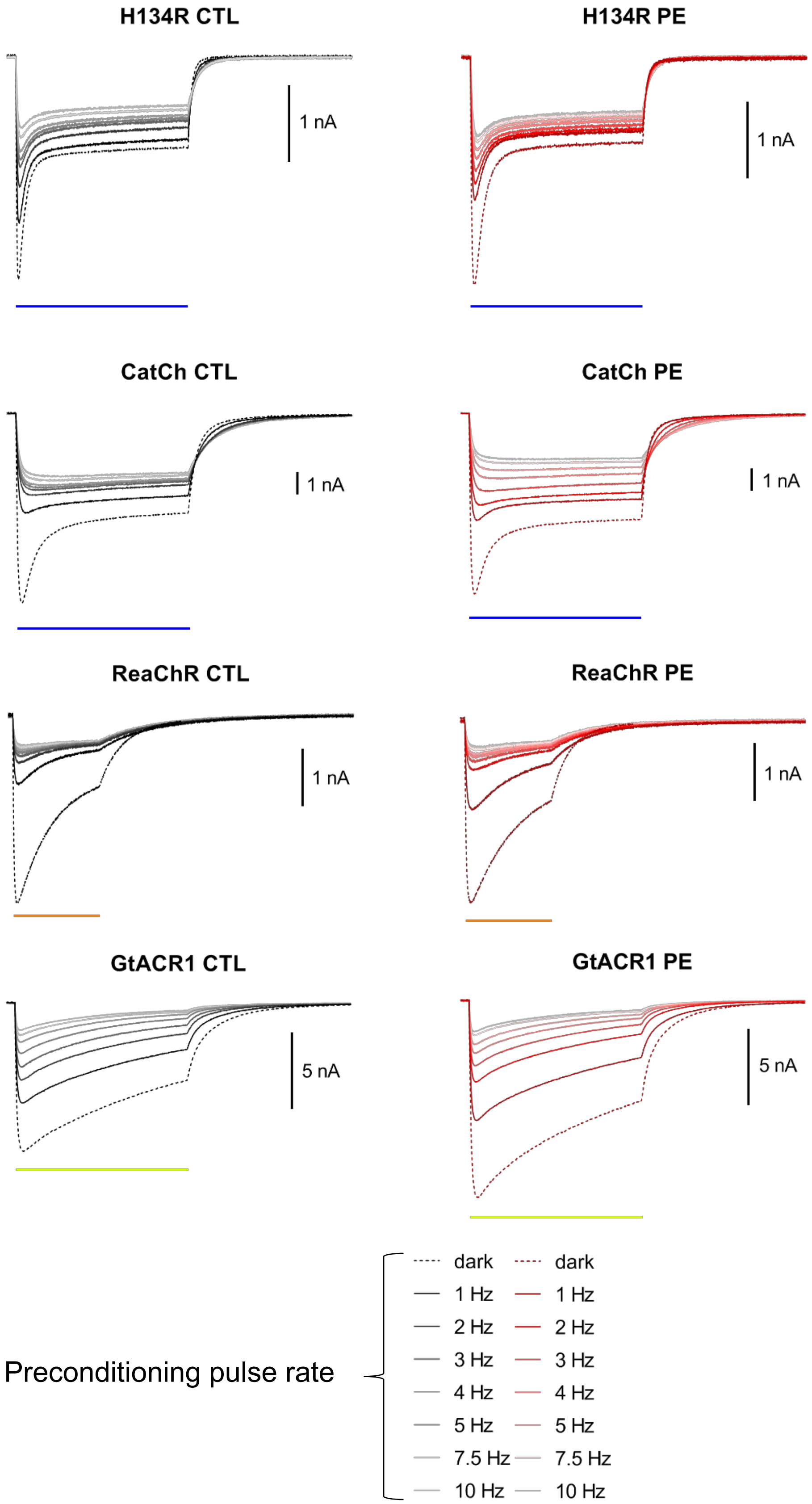

Supplementary Figure S5. Series of dark-adapted and preconditioned photocurrent traces evoked by 1-s blue (470 nm, for H134R and CatCh), amber (565 nm, for GtACR1) or red (617 nm, for ReaChR) light (1 mW/mm^2) exposure of CTL (black) and PE-treated (red) ChR-expressing NRVMs. Corresponding dark-adapted and preconditioned currents were recorded from the same cell and are shown overlaid.

Supplementary Figure S6

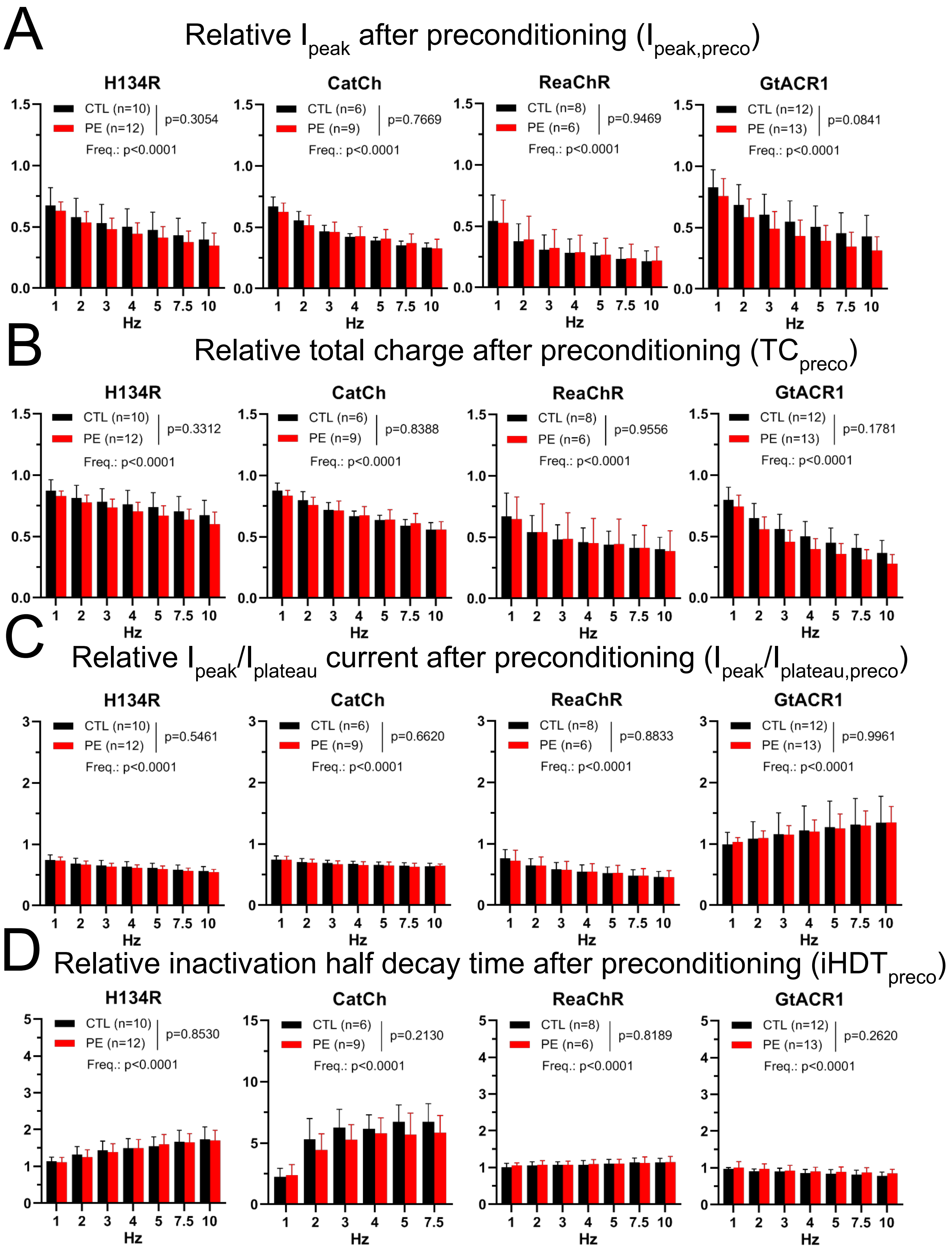

Supplementary Figure S6. Properties of photocurrents elicited, after preconditioning, by 1-s blue (470 nm, for H134R and CatCh), amber (565 nm, for GtACR1) or red (617 nm, for ReaChR) light pulses (1 mW/mm^2) in control (CTL) and in PE-treated (PE) ChR-expressing NRVMs. **A**: Preconditioned peak current amplitudes ($I_{peak,preco}$) relative to I_{peak} observed in the dark-adapted state. **B**: Total charge (TC) carried by preconditioned currents (TC_{preco}) relative to TC observed in the dark-adapted state. **C**: $I_{peak}/I_{plateau}$ ratios of preconditioned currents ($I_{peak}/I_{plateau,preco}$) relative to $I_{peak}/I_{plateau}$ measured in the dark-adapted state. **D**: Inactivation half decay times of preconditioned currents ($iHDT_{preco}$) normalized to iHDT values observed in the dark-adapted state. Data are presented as mean ± 95% CI for the number of cells indicated in each panel. The p values corresponding to the effect of PE treatment versus CTL conditions and the effect of preconditioning light pulse frequency (Freq.) were calculated by 2-way analysis of variance.

Supplementary Figure S7

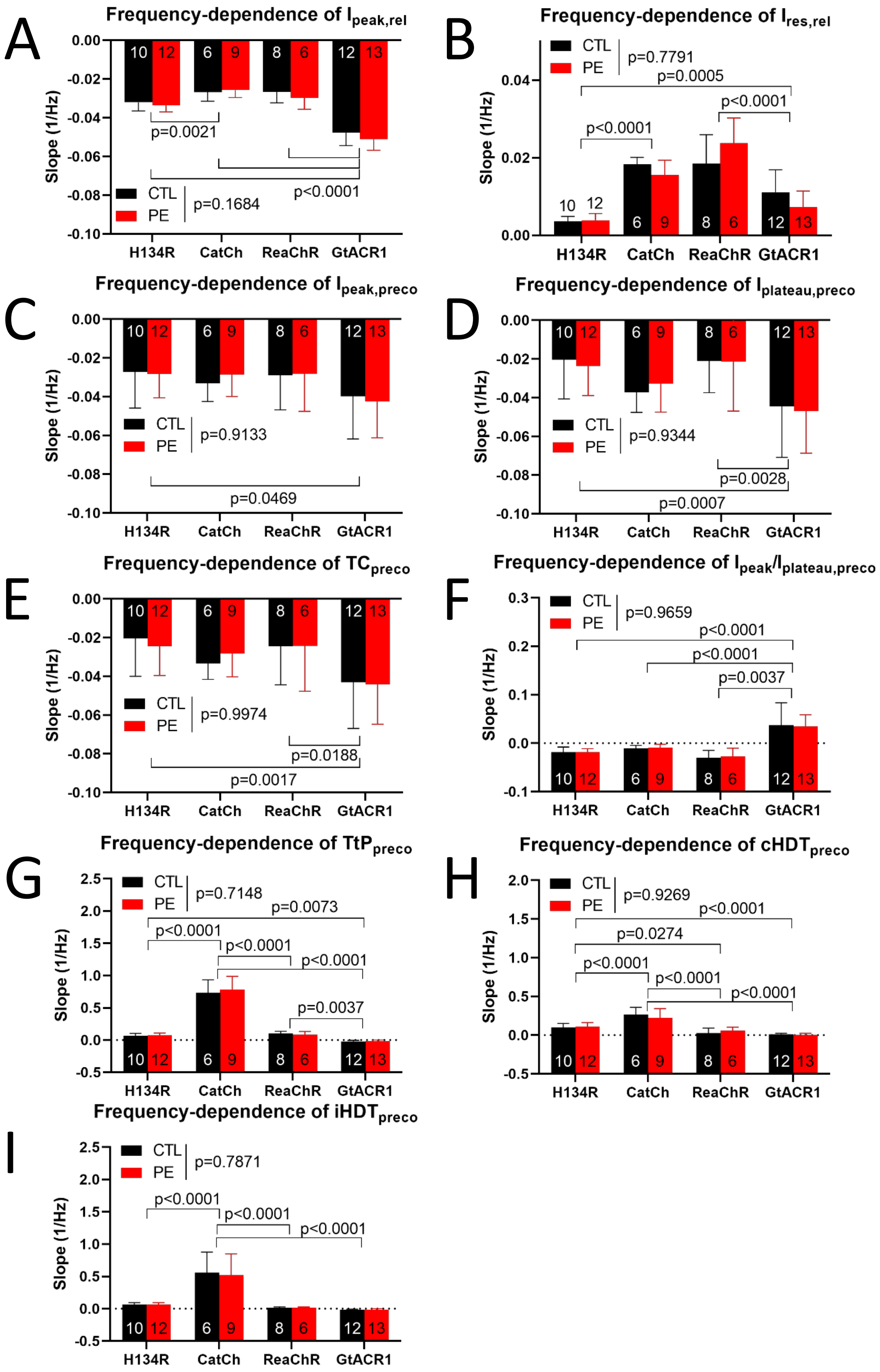

Supplementary Figure S7. Use-dependence of quantitative and kinetic parameters of ChR currents, as illustrated by mean ± standard deviation of trendline slopes. Trendline slopes were obtained by linear regression carried out on the data shown in the indicated figures, **A**: $I_{peak,rel}$ (**Figure 3B**), **B**: $I_{res,rel}$ (**Figure 3C**), **C**: $I_{peak,preco}$ (**Supplementary Material, Figure S6A**), **D**: $I_{plateau,preco}$ (**Figure 5B**), $I_{plateau,preco}$ (**Figure 5B**), **E**: TC_{preco} (**Supplementary Material, Figure S6B**), **F**: $I_{peak}/I_{plateau,preco}$ (**Supplementary Material, Figure S6C**), **G**: TtP_{preco} (**Figure 5C**), **H**: $iHDT_{preco}$ (**Supplementary Material, Figure S6D**), **I**: $cHDT_{preco}$ (**Figure 5D**). The p values corresponding to the effect of PE treatment versus CTL conditions and the effect of ChR variant on trendline slopes were calculated by 2-way analysis of variance followed by Tukey`s *post hoc* tests for multiple comparisons.

Supplementary Figure S8

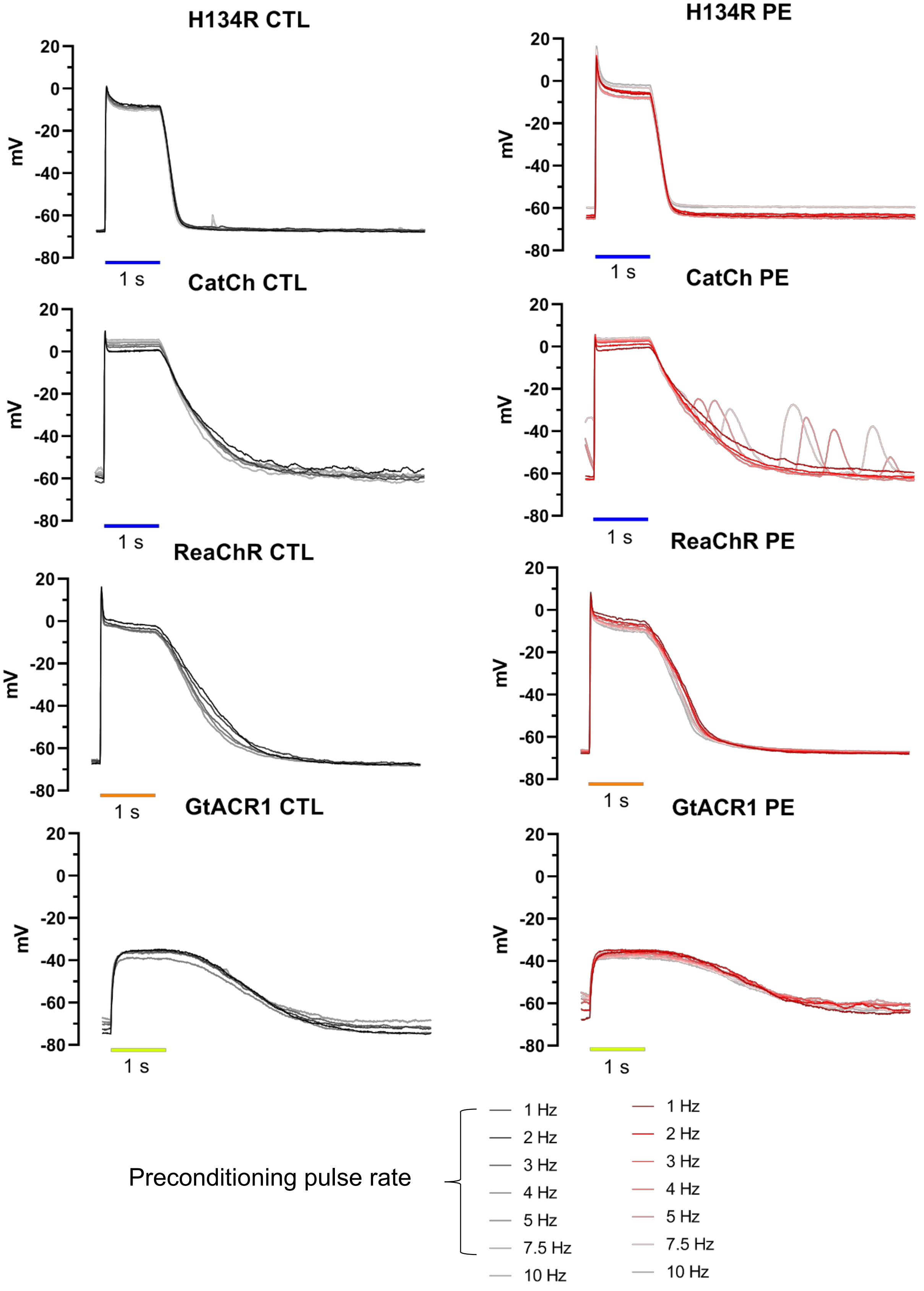

Supplementary Figure S8. Representative membrane potential recordings during 1-s illumination following preconditioning using blue (470 nm, for H134R and CatCh), amber (565 nm, for GtACR1) or red (617 nm, for ReaChR) light (1 mW/mm^2) in CTL (black) and in PE-treated (red) ChR-expressing NRVMs. Recordings obtained after 1, 2, 3, 4, 5, 7.5 and 10 Hz preconditioning are shown overlaid.

3 Supplementary Tables

Supplementary Table S1

Quantitative properties of preconditioned ChR currents. Data represent the average of relative values normalized to the same parameter observed in the dark-adapted state in the same cell. Graphical representations of these data are shown in **Figure 5** and **S6**.

$I_{peak,preco}$

	1 Hz	2 Hz	3 Hz	4 Hz	5 Hz	7.5 Hz	10 Hz
				**CTL**			
H134R	0.6738	0.5785	0.5307	0.5000	0.4739	0.4297	0.3972
CatCh	0.6691	0.5541	0.4655	0.4207	0.3915	0.3527	0.3338
ReaChR	0.5417	0.3775	0.3098	0.2823	0.2606	0.2316	0.2150
GtACR1	0.8271	0.6851	0.6035	0.5475	0.5038	0.4536	0.4269
				**PE**			
H134R	0.6302	0.5344	0.4809	0.4424	0.4121	0.3738	0.3470
CatCh	0.6247	0.5153	0.4603	0.4242	0.4052	0.3707	0.3275
ReaChR	0.5261	0.3913	0.3202	0.2876	0.2687	0.2376	0.2209
GtACR1	0.7552	0.5826	0.4894	0.4304	0.3926	0.3453	0.3142

$I_{plateau,preco}$

	1 Hz	2 Hz	3 Hz	4 Hz	5 Hz	7.5 Hz	10 Hz
				**CTL**			
H134R	0.8953	0.8328	0.8000	0.7781	0.7615	0.7224	0.6922
CatCh	0.8978	0.7818	0.6797	0.6303	0.5969	0.5497	0.5291

ReaChR	0.6960	0.5704	0.5157	0.4992	0.4862	0.4662	0.4540
GtACR1	0.7868	0.6212	0.5287	0.4652	0.4159	0.3648	0.3348
				**PE**			
H134R	0.8577	0.7971	0.7572	0.7207	0.6926	0.6577	0.6316
CatCh	0.8449	0.7443	0.6868	0.6477	0.6253	0.5924	0.5067
ReaChR	0.7215	0.5988	0.5453	0.5177	0.5047	0.4884	0.4791
GtACR1	0.7421	0.5445	0.4416	0.3773	0.3348	0.2859	0.2514

TC_{preco}

	**1 Hz**	**2 Hz**	**3 Hz**	**4 Hz**	**5 Hz**	**7.5 Hz**	**10 Hz**
				**CTL**			
H134R	0.8733	0.8137	0.7819	0.7601	0.7379	0.7041	0.6715
CatCh	0.8773	0.7987	0.7172	0.6663	0.6360	0.5907	0.5585
ReaChR	0.6686	0.5411	0.4811	0.4576	0.4388	0.4117	0.3991
GtACR1	0.7980	0.6478	0.5591	0.5000	0.4495	0.4041	0.3639
				**PE**			
H134R	0.8306	0.7364	0.6888	0.6528	0.6179	0.5752	0.5206
CatCh	0.8360	0.7571	0.7120	0.6741	0.6401	0.6109	0.5587
ReaChR	0.6454	0.5404	0.4847	0.4518	0.4443	0.4117	0.3844
GtACR1	0.7418	0.5575	0.4568	0.3951	0.3573	0.3123	0.2793

$I_{peak}/I_{plateau,preco}$

	**1 Hz**	**2 Hz**	**3 Hz**	**4 Hz**	**5 Hz**	**7.5 Hz**	**10 Hz**

CTL

H134R	0.7460	0.6850	0.6522	0.6317	0.6111	0.5834	0.5623
CatCh	0.7443	0.7060	0.6859	0.6714	0.6587	0.6440	0.6334
ReaChR	0.7660	0.6460	0.5857	0.5486	0.5224	0.4823	0.4615
GtACR1	0.9957	1.0823	1.1542	1.2180	1.2736	1.3116	1.3457

PE

H134R	0.7337	0.6681	0.6329	0.6114	0.5931	0.5650	0.5451
CatCh	0.7370	0.6915	0.6682	0.6539	0.6486	0.6273	0.6418
ReaChR	0.6190	0.5490	0.4950	0.5470	0.5233	0.4806	0.4551
GtACR1	1.0286	1.0964	1.1492	1.2010	1.2546	1.2972	1.3502

Supplementary Table S2

Kinetic properties of preconditioned ChR currents. Data represent the average of relative values normalized to the same parameter observed in the dark-adapted state in the same cell. Graphical representations of these data are shown in **Figure 5** and **S6**.

TtP_{preco}

	1 Hz	2 Hz	3 Hz	4 Hz	5 Hz	7.5 Hz	10 Hz
				**CTL**			
H134R	1.1768	1.3278	1.4415	1.5346	1.6325	1.7493	1.8265
CatCh	1.4564	2.3486	4.0668	5.7153	5.3568	7.0445	8.1969
ReaChR	1.1992	1.3421	1.4592	1.5472	1.6010	1.8301	2.1686
GtACR1	0.9940	0.9695	0.9083	0.8896	0.8580	0.8299	0.7970
				**PE**			
H134R	1.2089	1.4200	1.5474	1.6564	1.6717	1.8634	1.9238
CatCh	1.5930	2.5207	3.4813	4.4837	5.5685	6.7847	8.6693
ReaChR	1.1871	1.3781	1.5294	1.5327	1.5998	1.8556	1.9864
GtACR1	0.9861	0.9271	0.8858	0.8875	0.8469	0.8149	0.8220

$iHDT_{preco}$

	1 Hz	2 Hz	3 Hz	4 Hz	5 Hz	7.5 Hz	10 Hz
				**CTL**			
H134R	1.1410	1.3222	1.4320	1.4935	1.5458	1.6651	1.7351
CatCh	2.2532	5.3010	6.2733	6.1488	6.7315	6.7302	6.1220
ReaChR	1.0088	1.0543	1.0676	1.0707	1.1036	1.1350	1.1398

GtACR1	0.9675	0.9074	0.9038	0.8625	0.8462	0.8155	0.7866
				**PE**			
H134R	1.1117	1.2500	1.3862	1.4943	1.6000	1.6586	1.7094
CatCh	2.3862	4.4364	5.2678	5.7775	5.6772	5.8217	
ReaChR	1.0506	1.0700	1.0669	1.1006	1.1088	1.1216	1.1491
GtACR1	1.0010	0.9674	0.9310	0.9031	0.8973	0.8777	0.8527

$cHDT_{preco}$

	1 Hz	2 Hz	3 Hz	4 Hz	5 Hz	7.5 Hz	10 Hz
				**CTL**			
H134R	1.2332	1.4636	1.6089	1.7144	1.8305	2.0149	2.1147
CatCh	1.2440	1.9854	2.6989	3.0932	3.2916	3.6634	3.8177
ReaChR	1.6579	2.0257	2.1078	2.1863	2.1631	2.1523	2.0839
GtACR1	0.9856	0.9465	0.9480	0.9703	0.9783	0.9880	1.0478
				**PE**			
H134R	1.2287	1.4595	1.6582	1.8028	1.9378	2.0931	2.2945
CatCh	1.4666	2.1833	2.6781	2.9902	3.2723	3.4558	3.6798
ReaChR	1.3853	1.7210	1.8498	1.8948	1.9112	1.9822	2.0754
GtACR1	0.9982	0.9515	0.9570	0.9568	0.9647	0.9936	1.0017

4 Supplementary Methods

4.1 Immunocytochemical analysis of cell area and ANP expression

Cell area and ANP expression was quantified by immunocytochemical labeling of α-actinin and ANP as follows. NRVMs plated on bovine fibronectin (F1141, Sigma-Aldrich)-coated glass coverslips were fixed using pH-buffered 4% formaldehyde solution (Added Pharma) and permeabilized with 0.1% Triton X-100 in phosphate-buffered saline (PBS). For subsequent blocking of non-specific antibody binding, 0.1% normal donkey serum (D9663, Sigma-Aldrich) in PBS was used. Cells were then incubated with antibodies against α-actinin antibody (A7811, Sigma-Aldrich) and ANP (AB5490, Merck-Millipore) for 2 hours at room temperature, followed by incubation with Alexa 488-conjugated donkey anti-rabbit (A21206, Thermo Fisher Scientific) and Alexa 568-conjugated donkey anti-mouse (A10037, Thermo Fisher Scientific) secondary antibodies at room temperature for 45 minutes. Cell nuclei were counterstained with Hoechst 33342 (H3570, Thermo Fisher Scientific). Coverslips were mounted on a microscope slide using Vectashield (Vector Laboratories). Images were acquired by a Nikon Eclipse 80i microscope with a 10× objective for quantitative analysis and with a 40× objective for generating the representative images shown in Fig. S1A using NIS-Elements (v4.6, Nikon Instruments) software. Image analysis was done using the ImageJ (version 1.52p, National Institutes of Health, USA) software without any post-acquisition modification. Cell area and ANP expression was analysed in two NRVM cultures per treatment group using at least 5 images per culture and at least 5 (median for CTL: 12 and for PR: 17) cell per image. Cells showing α-actinin labeling were selected by using the ”Wand Tool” after customizing tool settings for each cell and visually verifying complete overlap between the selected area and the edges of the cell. Well-separated, single cells were preferred. Cell area was computed as the number of pixels within the selected

area. The same area was then selected in the corresponding image showing ANP labeling and the integral of all pixels resulting from the immunostaining of ANP within the selection area was calculated to quantify the ANP expression level. In case individual cells could not be selected separately, both cell area and ANP expression values were divided by the number of nuclei observed within the quantified area.

4.2 Total cellular protein content measurement

Lysates were prepared from six PE-treated and control (CTL) cultures each containing 3×10^5 cells using equal volumes of 150 mM NaCl, 50 mM Tris-HCl, 1% Triton X-100 and 0.5% sodium desoxycholate, 0.1% sodium dodecylsulphate (pH 8.0), supplemented with Complete, Mini, EDTA-free Protease Inhibitor Cocktail (Roche) according to the manufacturer`s instructions. The protein concentration in the cell lysates was measured by the Pierce BCA Protein Assay Kit (Thermo Fisher Scientific). Total cellular protein content was expressed in picograms (pg) per cells.

4.3 Patch clamp and light delivery

Patch clamp experiments were carried out on single, well-separated NRVMs using the perforated and ruptured patch clamp configuration for the recording of membrane potentials and transmembrane currents, respectively, as previously described (Liu et al., 2018). Briefly, NRVMs cultured on bovine fibronectin-coated glass coverslips were transferred into the patch clamp bath and were continuously superfused with (in mM): 126 NaCl, 5.4 KCl, 1 $MgCl_2$, 1.8 $CaCl_2$, 11 glucose, 10 HEPES-NaOH, pH 7.4. Borosilicate glass pipette electrodes were filled with (in mM): 81.1 L-aspartic acid, 110 KOH, 40 KCl, 5 K_2ATP, 0.1 GTP, 5 EGTA, 5 $MgCl_2$, 10 HEPES-KOH, pH 7.2 and 0.216 amphotericin-B. Channelrhodopsin (ChR)–expressing cardiomyocytes were identified with the aid of the fluorescent protein tags fused to their

carboxy-termini. After gigaseal formation, all light sources were switched off and the light-impermeant cover of the Faraday cage was closed to ensure a completely dark environment. A dark period was maintained for a minimum of 3 minutes before the initiation of any illumination protocol. Membrane potential recordings were obtained in the perforated whole cell configuration with series resistances < 50 MΩ. Action potentials (APs) were triggered by 2- to 10-ms current pulses with suprathreshold amplitudes at 1 Hz. In a typical experiment, following completion of membrane potential recordings, the ruptured whole cell configuration was established by gentle suction and in case intact gigaseal could be confirmed, ChR currents were recorded in voltage clamp mode using matching illumination protocols as follows. Membrane potential was clamped at -90 mV and the series resistance (5.5 ± 4.9 MΩ) was compensated electronically to >80%. Cell membrane capacitance was estimated by dividing the time constant of the decay of the capacitive transient in response to 10 mV hyperpolarizing voltage clamp steps from −10 mV by the series resistance. Membrane potential and command potential values were corrected *post hoc* by the empirically determined liquid junction potential (-10 mV). Patch clamp experiments were carried out at room temperature (23-25°C).

NRVMs were illuminated by using 470- (M470L3-C4), 565- (M565L3) or 617-nm (M617L3) light-emitting diodes (LEDs) mounted on COP1-A collimation lenses (all from Thorlabs) on three independent camera ports of a Zeiss Axiovert 35 inverted microscope via a 40× magnification objective. LEDs were driven by LEDD1B driver units (Thorlabs) controlled by the Clampex software (v10, Molecular Devices) using independent analog out ports of the Digidata 1440 D/A interface (Molecular Devices). The intensity of the light released by the objective lens was measured by using a PM100D power meter equipped with a S130C photodiode probe (both from Thorlabs), using factory-set corrections for each wavelength. The same illumination

intensity of 1 mW/mm^2 was used for all wavelengths and in all experiments. The delay between peak driver voltage and peak light intensity was <0.2 ms. The combined light transmission of the glass bottom of the patch clamp bath and the glass coverslip on which the NRVMs were cultured was 84%.

Dark-adapted ChRs were activated by a 1-s light pulse delivered following a 3-minute dark period. Graded light adaptation was achieved by the application of a preconditioning illumination protocol, consisting of 30-s trains of 10-ms light pulses at frequencies increasing from 1 to 10 Hz. The 1-Hz pulse train was initiated after a dark period of 3 minutes and each pulse train was followed by a 1-s light pulse to obtain matching parameters from ChRs in dark- and light-adapted states.

4.4 Data analysis

Numerical data were extracted from binary ABF files by using Python 3.7 and the pyABF module[1] and they were analyzed with custom software. Current traces were smoothed by locally weighted scatterplot smoothing and were visually inspected before analysis. Baseline currents were calculated by averaging the current during the 10 ms preceding onset of a single light pulse or of the first light pulse in a preconditioning light pulse train and were subtracted from all current amplitudes. For recordings with 1-s illumination periods, peak current (I_{peak}) was defined as the extremum between light onset and offset while plateau current ($I_{plateau}$) and plateau potential ($V_{plateau}$) were measured at light offset. Time to I_{peak} (TtP) was determined by measuring the time interval between light onset and I_{peak}, inactivation half-decay time (iHDT) was computed as time between I_{peak} and 50% of the inactivation component (*i.e.* I_{peak} minus $I_{plateau}$) and closing half decay time (cHDT) was defined as the time from light offset to 50% of $I_{plateau}$.

---

[1] Harden, S.W. (2020). pyABF 2.2.3. https://pypi.org/project/pyabf/

Baseline-corrected current traces were integrated to obtain total charges. Current and charge densities were calculated by normalizing current amplitudes and total charges by the cells' capacitances. During the preconditioning illumination protocol for each pulse train, I_{peak} was defined as the extremum between the two light onsets. Residual currents (I_{res}) were measured within 1 ms before light onset. To assess preconditioning-induced changes of I_{peak} and I_{res}, the last I_{peak} and I_{res} values of each pulse train were normalized by the first I_{peak} of the 1-Hz pulse train measured in the same cell, yielding $I_{peak,rel}$ and $I_{plateau,rel}$. Following each preconditioning pulse train, a 1-s light pulse was delivered. Quantitative and kinetic properties of ChR current evoked by this 1-s illumination were normalized to the corresponding dark-adapted values obtained from the same cell, yielding $I_{peak,preco}$, $I_{plateau,preco}$, TtP_{preco}, $iHDT_{preco}$, $cHDT_{preco}$, TC_{preco} and $I_{peak}/I_{plaeau,preco}$. The membrane potential (V_m) response to dark-adapted ChR activation was assessed by measuring membrane potential at light offset ($V_{plateau}$). APs were characterized by AP duration until 80% repolarization (APD_{80}), defined as time between trigger pulse onset to 80% of AP amplitude (peak V_m minus baseline). The V_m response during preconditioning was quantified by measuring V_{peak}, defined as maximal V_m between the last two light onsets and V_{res}, defined as V_m within 1 ms before the last light onset of each pulse train. The V_m response to 1-s illumination following preconditioning was characterized by $V_{plateau}$, which was measured at light offset and designated $V_{plateau,preco}$.

5 References

Askar, S.F., Ramkisoensing, A.A., Schalij, M.J., Bingen, B.O., Swildens, J., Van Der Laarse, A., Atsma, D.E., De Vries, A.A., Ypey, D.L., and Pijnappels, D.A. (2011). Antiproliferative treatment of myofibroblasts prevents arrhythmias in vitro by limiting myofibroblast-induced depolarization. *Cardiovasc Res* 90**,** 295-304.

Liu, J., Volkers, L., Jangsangthong, W., Bart, C.I., Engels, M.C., Zhou, G., Schalij, M.J., Ypey, D.L., Pijnappels, D.A., and De Vries, A.a.F. (2018). Generation and primary characterization of iAM-1, a versatile new line of conditionally immortalized atrial myocytes with preserved cardiomyogenic differentiation capacity. *Cardiovasc Res* 114**,** 1848-1859.
